# Multi-omics analysis of glucose-mediated signaling by a moonlighting Gβ protein Asc1/RACK1

**DOI:** 10.1371/journal.pgen.1009640

**Published:** 2021-07-02

**Authors:** Shuang Li, Yuanyuan Li, Blake R. Rushing, Sarah E. Harris, Susan L. McRitchie, Janice C. Jones, Daniel Dominguez, Susan J. Sumner, Henrik G. Dohlman

**Affiliations:** 1 Department of Pharmacology, University of North Carolina at Chapel Hill, Chapel Hill, North Carolina, United States of America; 2 Nutrition Research Institute, Department of Nutrition, School of Public Health, University of North Carolina at Chapel Hill, Chapel Hill, North Carolina, United States of America; 3 Department of Biochemistry and Biophysics, University of North Carolina at Chapel Hill, Chapel Hill, North Carolina, United States of America; Pacific Northwest Research Institute, UNITED STATES

## Abstract

Heterotrimeric G proteins were originally discovered through efforts to understand the effects of hormones, such as glucagon and epinephrine, on glucose metabolism. On the other hand, many cellular metabolites, including glucose, serve as ligands for G protein-coupled receptors. Here we investigate the consequences of glucose-mediated receptor signaling, and in particular the role of a Gα subunit Gpa2 and a non-canonical Gβ subunit, known as Asc1 in yeast and RACK1 in animals. Asc1/RACK1 is of particular interest because it has multiple, seemingly unrelated, functions in the cell. The existence of such “moonlighting” operations has complicated the determination of phenotype from genotype. Through a comparative analysis of individual gene deletion mutants, and by integrating transcriptomics and metabolomics measurements, we have determined the relative contributions of the Gα and Gβ protein subunits to glucose-initiated processes in yeast. We determined that Gpa2 is primarily involved in regulating carbohydrate metabolism while Asc1 is primarily involved in amino acid metabolism. Both proteins are involved in regulating purine metabolism. Of the two subunits, Gpa2 regulates a greater number of gene transcripts and was particularly important in determining the amplitude of response to glucose addition. We conclude that the two G protein subunits regulate distinct but complementary processes downstream of the glucose-sensing receptor, as well as processes that lead ultimately to changes in cell growth and metabolism.

## Author summary

Despite the societal importance of glucose fermentation in yeast, the mechanisms by which these cells detect and respond to glucose have remained obscure. Glucose detection requires a cell surface receptor coupled to a G protein that is comprised of two subunits, rather than the more typical heterotrimer: an α subunit Gpa2 and the β subunit Asc1 (or RACK1 in humans). Asc1/RACK1 also serves as a subunit of the ribosome, where it regulates the synthesis of proteins involved in glucose fermentation. This manuscript uses global metabolomics and transcriptomics to demonstrate the distinct roles of each G protein subunit in transmitting the glucose signal. Whereas Gpa2 is primarily involved in the metabolism of carbohydrates, Asc1/RACK1 contributes to production of amino acids necessary for protein synthesis and cell division. These findings reveal the initial steps of glucose signaling and several unique and complementary functions of the G protein subunits. More broadly, the integrated approach used here is likely to guide efforts to determine the topology of complex G protein and metabolic signaling networks in humans.

## Introduction

All cells respond to changes in extracellular and environmental conditions, and many of these inputs are detected by receptors coupled to guanine nucleotide-binding proteins (G proteins). While G protein-coupled receptors (GPCRs) have established roles in detecting odors, light, hormones, and neurotransmitters, more recent investigations have uncovered an important role for GPCRs in responding to nutrients and metabolites such as glucose, amino acids, purine nucleotides, and carboxylic acids including fatty acids [1]. GPCR activation leads to the synthesis of chemical second messengers, changes in cell metabolism and transcriptional reprograming. Thus, G proteins act as signal transducers, transmitting a specific extracellular signal to a variety of intracellular second messengers and chemical metabolites. In some cases, the initiating and ensuing signals are one and the same.

The yeast *Saccharomyces cerevisiae* has two G protein signaling systems, one that responds to mating pheromone and another that responds to glucose. These systems do not share components but appear to act in a coordinated fashion; when glucose is limiting, the mating response is delayed until the cells have undergone two complete rounds of cell division [2]. Of these GPCR systems, the pheromone pathway is the best characterized and is typical of those found in humans. A peptide ligand binds to a cell surface receptor, which then activates a heterotrimeric G protein, comprised of an α subunit and a tightly associated βγ subunit dimer. Gα then exchanges GDP for GTP and dissociates from Gβγ. The Gα subunit activates a phosphatidylinositol 3-kinase while Gβγ initiates a mitogen-activated protein kinase (MAPK) cascade [3,4].

The second GPCR pathway responds to glucose [5]. The presumptive glucose receptor (Gpr1) is coupled to a typical Gα (Gpa2) [6–8], but there is no corresponding Gβγ. Rather, Gpa2 appears to assemble with a multifunctional protein known as RACK1 in animals [9] Gib2 in *Cryptococcus neoformans* [10,11], and Asc1 in *Saccharomyces cerevisiae* [12]. Whereas Gpa2 activates adenylyl cyclase, leading to a transient increase in cellular cAMP [13], Asc1 has the opposite effect on cAMP production [12]. It is also common in animal cells that Gα and Gβγ bind to and regulate the same effector enzymes, including adenylyl cyclase, in opposition to one another [14].

While Asc1/RACK1 has characteristics of a Gβ subunit, it also has other important functions in the cell. RACK1 was originally identified as an adaptor for protein kinase C in animals, and was proposed to have a role in kinase-mediated signal transduction [15]. RACK1 has also been demonstrated to interact with several GPCRs and G protein βγ subunits [16–19]. Most prominently, Asc1/RACK1 is part of the 40S subunit of the ribosome [20–25]. In that capacity, Asc1 plays an important role in recruiting quality control systems that diminish frameshifting errors when translation is stalled [26,27]. Moreover, Asc1 regulates a subset of transcripts primarily related to glycolysis, respiration, oxidative stress and fermentation [28]. Thus Asc1 is part of two distinct molecular complexes, one involved in glucose sensing and the other in glucose utilization. While the function of Asc1 in the ribosome has been well characterized, its role as a G protein is largely unexplored.

Here, we sought to determine the role of Asc1, in comparison with Gpa2, to glucose signaling. To gain a better understanding of their relative contributions to cell physiology, we undertook an integrated metabolomics and transcriptomics analysis, comparing the glucose response in mutants that lack the Gα or Gβ subunit. By this approach, one that is largely unprecedented in the GPCR field, we have identified the earliest events leading to glucose fermentation. Our analysis is likely to guide similar efforts to determine the topology of complex G protein and metabolic signaling networks in humans.

## Results

We determined previously that Asc1 binds to Gpa2, that these proteins have opposing effects on adenylyl cyclase activity, and that they act in response to the glucose receptor Gpr1 [12]. Here we sought a deeper understanding of the molecular and cellular consequences of G protein activation. To that end, we undertook a multi-platform investigation, performing untargeted metabolomics and transcriptomics analysis, in cells lacking each of these proteins, in response to glucose. By measuring changes in gene expression and perturbations in host metabolism we sought to gain an understanding of Asc1, apart from its role in translation, and how it complements the functions of Gpa2.

Wildtype, *gpr1*, *gpa2* and *asc1* cells (all without nutritional auxotrophic markers) were grown for 1 h in low (L, 0.05%) glucose, then divided and either left untreated or treated with high (H, 2%) glucose for 2 minutes (metabolomics) or 10 minutes (transcriptomics). These time points were selected based on prior data, showing an early and transient spike of cAMP and a subsequent induction of genes within 10 minutes of glucose treatment (see Methods) [29]. We then analyzed our data using Principal Component Analysis (PCA). This unsupervised multivariate analysis method is particularly useful for the visualization of the relationship between observations and variables. When applied to our transcriptomics data, PCA indicated good differentiation of groups based on the proximity of data points for a given treatment and genotype (S1A Fig). This analysis revealed that PC1, which aligns primarily with treatment, accounts for 90% of variance while PC2, which aligns primarily with genotype, represents 5% of variance. Thus the first 2 components explained 95% of the variance. For metabolomics, the first 2 components explained 63% of the variance (S1B Fig). For both measurements, and as expected, *gpr1* aligned closely with *gpa2* [6–8,12,13]. Both measures are consistent with previously established opposing effects of Gα and Gβ on processes downstream of the G protein.

**Fig 1:**
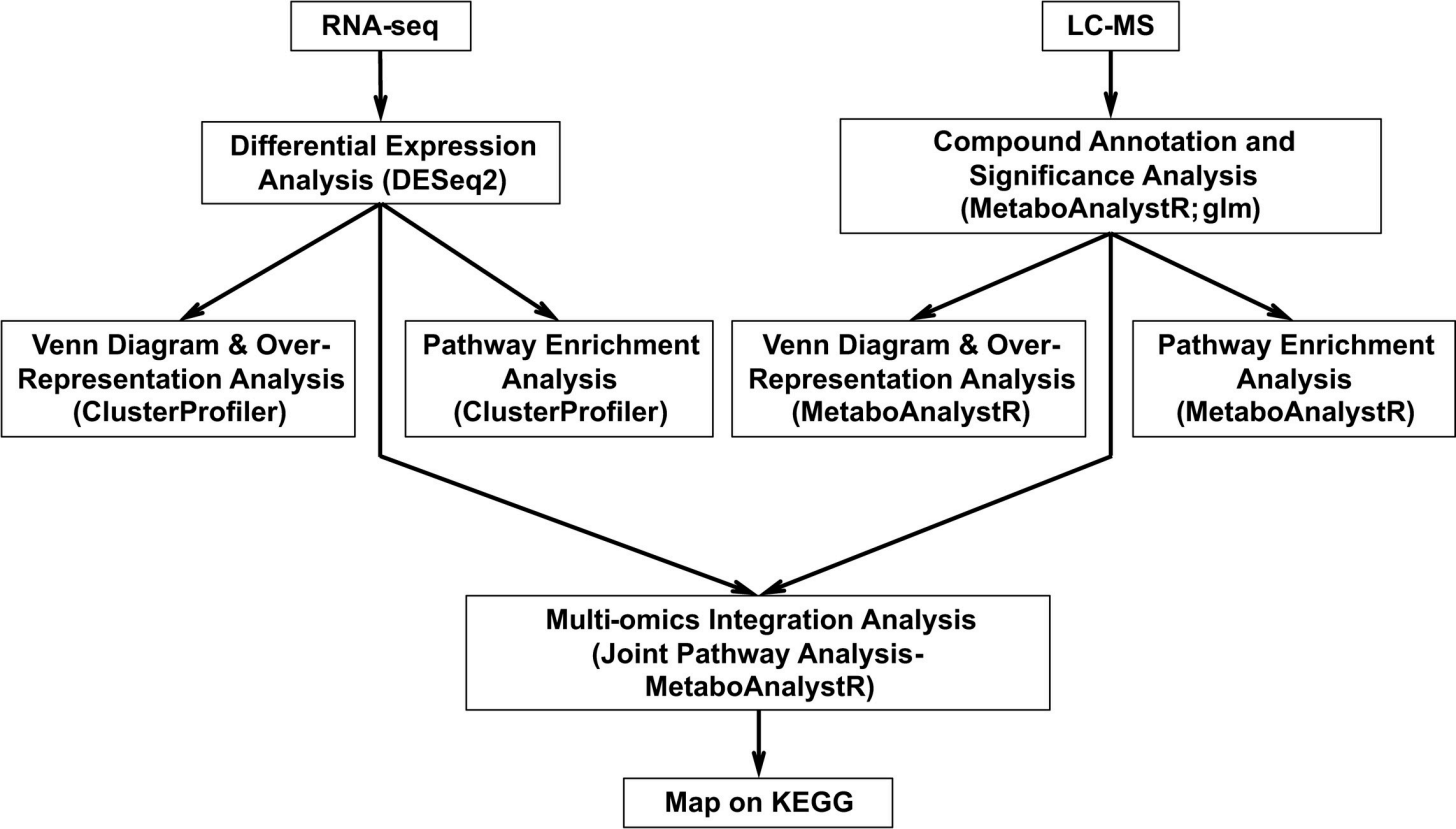
Analysis pipeline. Workflow of concentration analysis and sensitivity analysis for transcriptomics and metabolomics data.

Our next objective was to identify the specific pathways and processes regulated by each G protein subunit. To that end, we analyzed the transcriptomics and metabolomics data, with and without glucose addition, for wildtype, *gpr1*, *gpa2* and *asc1* cells. Herein we use the term “concentration analysis” when comparing the data for mutant and wildtype cells after the addition of glucose (Figs 1 and S2A), and we use the term “sensitivity analysis” when comparing the difference in response at high and low glucose for the mutants (mutantH-mutantL) and the wildtype (wtH-wtL) cells (Figs 1 and S2B). Thus, concentration analysis reveals cell status after glucose addition, while sensitivity analysis reveals response amplitude (H-L) differences for the different strains. Such amplitude differences are important when a negative regulator restores the system to baseline in the face of sustained (or step-wise) activation by a positive regulator.

In summary, we present integrated transcription data and untargeted metabolomics data obtained for the cell extracts, as follows:

Concentration Analysis

- *gpa2* mutant vs. wildtype at high (2%) glucose
- *asc1* mutant vs. wildtype at high (2%) glucose

We then compare the results of A and B, calculated as detailed in Methods.

Sensitivity Analysis

- difference between *gpa2* mutant at high and low glucose (gpa2H-gpa2L) vs. difference between wildtype at high and low glucose (wtH-wtL)
- difference between *asc1* mutant at high and low glucose (asc1H-asc1L) vs. difference between wildtype at high and low glucose (wtH-wtL)

We then compare the results of A and B, calculated as detailed in Methods.

### Concentration analysis

We began by establishing the transcriptional and metabolic profile of wildtype cells. We defined the differentially-expressed genes (DEGs) as having an adjusted p-value <0.05, absolute log2 fold-change value >1 and baseMean >100. Then, using the ClusterProfiler package in R [30] we performed gene set enrichment analysis (GSEA) in the Kyoto Encyclopedia of Genes and Genomes (KEGG) [31–33]. This database provides an overview of biological pathways in the cell, as determined by genome sequencing and other high-throughput methods. GSEA determines whether a defined set of genes shows statistically significant and concordant differences between two phenotypes. Since it is a rank-based analysis no cutoff is needed (see Tables 1–5, ‘Transcriptomics’).

**Table 1:** Single-omics analysis results for wildtype comparing 2% (H) and 0.05% (L) glucose. First block shows GSEA for transcriptomics with adjusted p-value <0.05, arranged in ascending order; second block shows MetaboAnalystR pathway enrichment analysis for metabolomics with combined p-value <0.05 arranged in ascending order, as detailed in Methods. Enriched pathways refer to classifications provided in KEGG.

Transcriptomics				Metabolomics	
enriched pathways	adjusted p-value	enriched pathways	adjusted p-value	enriched pathways	combined p-value
Ribosome	0.0023	Lysine biosynthesis	0.0051	Starch and sucrose metabolism	0.0010
Cell cycle—yeast	0.0023	Fructose and mannose metabolism	0.0075	Tyrosine metabolism	0.0065
Biosynthesis of amino acids	0.0023	Aminoacyl-tRNA biosynthesis	0.0080	Galactose metabolism	0.0073
RNA transport	0.0023	Sulfur metabolism	0.0094	Cysteine and methionine metabolism	0.0184
Ribosome biogenesis in eukaryotes	0.0023	One carbon pool by folate	0.0098	Glycolysis / Gluconeogenesis	0.0198
Cysteine and methionine metabolism	0.0023	Valine, leucine and isoleucine biosynthesis	0.0101	Inositol phosphate metabolism	0.0207
RNA polymerase	0.0023	Proteasome	0.0101	Amino sugar and nucleotide sugar metabolism	0.0233
Purine metabolism	0.0028	Autophagy—yeast	0.0184	Purine metabolism	0.0343
DNA replication	0.0028	Pyrimidine metabolism	0.0184	N-Glycan biosynthesis	0.0361
Galactose metabolism	0.0028	Amino sugar and nucleotide sugar metabolism	0.0188	Pentose phosphate pathway	0.0364
Glyoxylate and dicarboxylate metabolism	0.0028	Selenocompound metabolism	0.0226	Butanoate metabolism	0.0443
Citrate cycle (TCA cycle)	0.0028	Mismatch repair	0.0231		
Peroxisome	0.0028	Glycine, serine and threonine metabolism	0.0322		
Starch and sucrose metabolism	0.0028	Glycolysis / Gluconeogenesis	0.0387		
Oxidative phosphorylation	0.0033	Autophagy—other	0.0416		
Carbon metabolism	0.0041	Meiosis—yeast	0.0474		

**Table 2:** Single- and multi-omics integration results for *gpa2* by concentration analysis. First block shows GSEA for transcriptomics with adjusted p-value <0.05, arranged in ascending order; second block shows MetaboAnalystR pathway enrichment analysis for metabolomics with combined p-value <0.05, arranged in ascending order; third block shows MetaboAnalystR joint pathway analysis with adjusted p-value <0.05, arranged in ascending order, as detailed in Methods.

Transcriptomics		Metabolomics		Integration	
enriched pathways	adjusted p-value	enriched pathways	combined p-value	enriched pathways	adjusted p-value
Ribosome biogenesis in eukaryotes	0.0084	Purine metabolism	0.0021	Oxidative phosphorylation	3.13E-14
Oxidative phosphorylation	0.0084	Fructose and mannose metabolism	0.0047	Galactose metabolism	1.60E-11
		Amino sugar and nucleotide sugar metabolism	0.0079	ABC transporters	1.31E-08
		Galactose metabolism	0.0079	Glycolysis or Gluconeogenesis	8.02E-05
		Glutathione metabolism	0.0155	Fructose and mannose metabolism	8.18E-05
		Tyrosine metabolism	0.0224	Starch and sucrose metabolism	8.18E-05
		Arginine biosynthesis	0.0234	Arginine biosynthesis	0.0012
		Biotin metabolism	0.0252	Pentose phosphate pathway	0.0012
		Aminoacyl-tRNA biosynthesis	0.0360	Purine metabolism	0.0017
		Starch and sucrose metabolism	0.0396	Amino sugar and nucleotide sugar metabolism	0.0073
		Phosphatidylinositol signaling system	0.0424	beta-Alanine metabolism	0.0119
				Alanine, aspartate and glutamate metabolism	0.0139
				Cysteine and methionine metabolism	0.0149
				Citrate cycle (TCA cycle)	0.0221

**Table 3:** Single- and multi-omics integration results for *asc1* by concentration analysis. First block shows GSEA for transcriptomics with adjusted p-value <0.05, arranged in ascending order; second block shows MetaboAnalystR pathway enrichment analysis for metabolomics with combined p-value <0.05, arranged in ascending order; third block shows MetaboAnalystR joint pathway analysis with adjusted p-value <0.05, arranged in ascending order, as detailed in Methods.

Transcriptomics		Metabolomics		Integration	
enriched pathways	adjusted p-value	enriched pathways	combined p-value	enriched pathways	adjusted p-value
Starch and sucrose metabolism	0.0155	Arginine biosynthesis	0.0029	Arginine biosynthesis	7.47E-10
Biosynthesis of amino acids	0.0156	Glutathione metabolism	0.0054	ABC transporters	7.47E-10
Sulfur metabolism	0.0156	Aminoacyl-tRNA biosynthesis	0.0214	Purine metabolism	1.41E-09
Ribosome	0.0156	Purine metabolism	0.0481	Tryptophan metabolism	0.0074
Galactose metabolism	0.0156			Galactose metabolism	0.0074
Meiosis—yeast	0.0156			Cysteine and methionine metabolism	0.0087
Glycine, serine and threonine metabolism	0.0243			Alanine, aspartate and glutamate metabolism	0.0087
One carbon pool by folate	0.0243			Histidine metabolism	0.0087
Aminoacyl-tRNA biosynthesis	0.0243			Arginine and proline metabolism	0.0136
2-Oxocarboxylic acid metabolism	0.0387			Phenylalanine metabolism	0.0191
Lysine biosynthesis	0.0477			Lysine biosynthesis	0.0348
Ribosome biogenesis in eukaryotes	0.0477			Amino sugar and nucleotide sugar metabolism	0.0465

**Table 4:** Single- and Multi-omics integration results for *gpa2* by sensitivity analysis. First block shows GSEA for transcriptomics with adjusted p-value <0.05, arranged in ascending order; second block shows MetaboAnalystR pathway enrichment analysis for metabolomics with combined p-value <0.05, arranged in ascending order; third block shows MetaboAnalystR joint pathway analysis with adjusted p-value <0.05, arranged in ascending order, as detailed in Methods.

Transcriptomics		Metabolomics		Integration	
enriched pathways	adjusted p-value	enriched pathways	combined p-value	enriched pathways	adjusted p-value
N-Glycan biosynthesis	0.0050	Fructose and mannose metabolism	0.0044	Glycolysis or Gluconeogenesis	2.32E-06
Various types of N-glycan biosynthesis	0.0050	Glycolysis / Gluconeogenesis	0.0183	Galactose metabolism	2.32E-06
DNA replication	0.0050	Methane metabolism	0.0211	Cell cycle	1.61E-05
Aminoacyl-tRNA biosynthesis	0.0050	Porphyrin and chlorophyll metabolism	0.0473	Pentose phosphate pathway	2.03E-05
Cysteine and methionine metabolism	0.0050			Starch and sucrose metabolism	3.41E-05
Starch and sucrose metabolism	0.0050			MAPK signaling pathway	0.0002
Galactose metabolism	0.0050			Meiosis	0.0010
Cell cycle	0.0056			Fructose and mannose metabolism	0.0010
Mismatch repair	0.0063			Purine metabolism	0.0025
Steroid biosynthesis	0.0308			Cysteine and methionine metabolism	0.0025
				Protein processing in endoplasmic reticulum	0.0096
				Peroxisome	0.0124
				Spliceosome	0.0230
				Pyruvate metabolism	0.0238
				DNA replication	0.0238
				Longevity regulating pathway	0.0238
				Glycerolipid metabolism	0.0238
				beta-Alanine metabolism	0.0238
				Steroid biosynthesis	0.0238
				Amino sugar and nucleotide sugar metabolism	0.0238
				Alanine, aspartate and glutamate metabolism	0.0238
				Inositol phosphate metabolism	0.0241

**Table 5:** Single- and Multi-omics integration results for *asc1* by sensitivity analysis. First block shows GSEA for transcriptomics with adjusted p-value <0.05, arranged in ascending order; second block shows MetaboAnalystR pathway enrichment analysis for metabolomics with combined p-value <0.05, arranged in ascending order; third block shows MetaboAnalystR joint pathway analysis with adjusted p-value <0.05, arranged in ascending order, as detailed in Methods.

Transcriptomics		Metabolomics		Integration	
enriched pathways	adjusted p-value	enriched pathways	combined p-value	enriched pathways	adjusted p-value
Ribosome	0.0071	Lysine degradation	0.0082	Ribosome	4.76E-98
Ribosome biogenesis in eukaryotes	0.0071	Cysteine and methionine metabolism	0.0473	Ribosome biogenesis in eukaryotes	1.87E-21
Glyoxylate and dicarboxylate metabolism	0.0071			RNA polymerase	0.0015
RNA polymerase	0.0071			Galactose metabolism	0.0015
Peroxisome	0.0071			Pentose phosphate pathway	0.0422
Galactose metabolism	0.0104			Purine metabolism	0.0422
Citrate cycle (TCA cycle)	0.0141			Arginine biosynthesis	0.0422
RNA transport	0.0196				
Starch and sucrose metabolism	0.0241				
Carbon metabolism	0.0479				
Propanoate metabolism	0.0479				
Aminoacyl-tRNA biosynthesis	0.0498				

When comparing wildtype before and after glucose addition (0.05% vs. 2%), we observed transcriptomic changes in 32 pathways, including ribosome, DNA replication, transcription, cell cycle as well as carbon, amino acids, lipids and nucleotide metabolism (Table 1). These differences were expected, and reflect processes needed to transition from a low glucose phase where metabolism supports cellular homeostasis (e.g. autophagy, addressing reactive oxygen species, maintaining osmotic balance) to a high glucose phase where metabolism supports cell growth and division (structural rearrangements as well as anabolic processes to make building blocks that support cell proliferation) [34].

We next conducted untargeted metabolomics by mass spectrometry. Pathway enrichment analysis was performed in MetaboAnalystR [31,32], using the Fisher’s method to integrate Mummichog [33] and GSEA results to produce the combined p-values reported here (Table 1). The pathway enrichment analysis module Mummichog is optimized for detecting prominent changes while GSEA excels at detecting concordant small changes in peak intensity. Because of the uncertainty associated with peak annotation for LC-MS data, the reliability of pathway enrichment is improved when combining the results from two different statistical methods [31,32]. When comparing 0.05% to 2% glucose in wildtype, 11 pathways were perturbed with a combined p-value <0.05 (Table 1). These include perturbations in the metabolism of carbohydrates, amino acids, nucleotides and lipids, and are concordant with changes in gene transcription. Again, these differences reflect processes needed to prepare the cell for growth and division. In addition, they are likely related to the role of Asc1 and Gpa2 in haploid invasive growth [12,35,36], a process where cells form long branching filaments and exhibit increased adherence and invasion of the substratum during periods of glucose limitation [37].

We next considered the effects of glucose in each of the mutant strains and how each mutant compares with wildtype. Based on GSEA, the *gpa2* mutant exhibited changes in transcripts linked to oxidative phosphorylation and ribosome biogenesis (Tables 2 and S1). The *asc1* mutant was enriched for twelve pathways including ribosome biogenesis and metabolism of amino acids (Tables 3 and S2).

**Fig 2:**
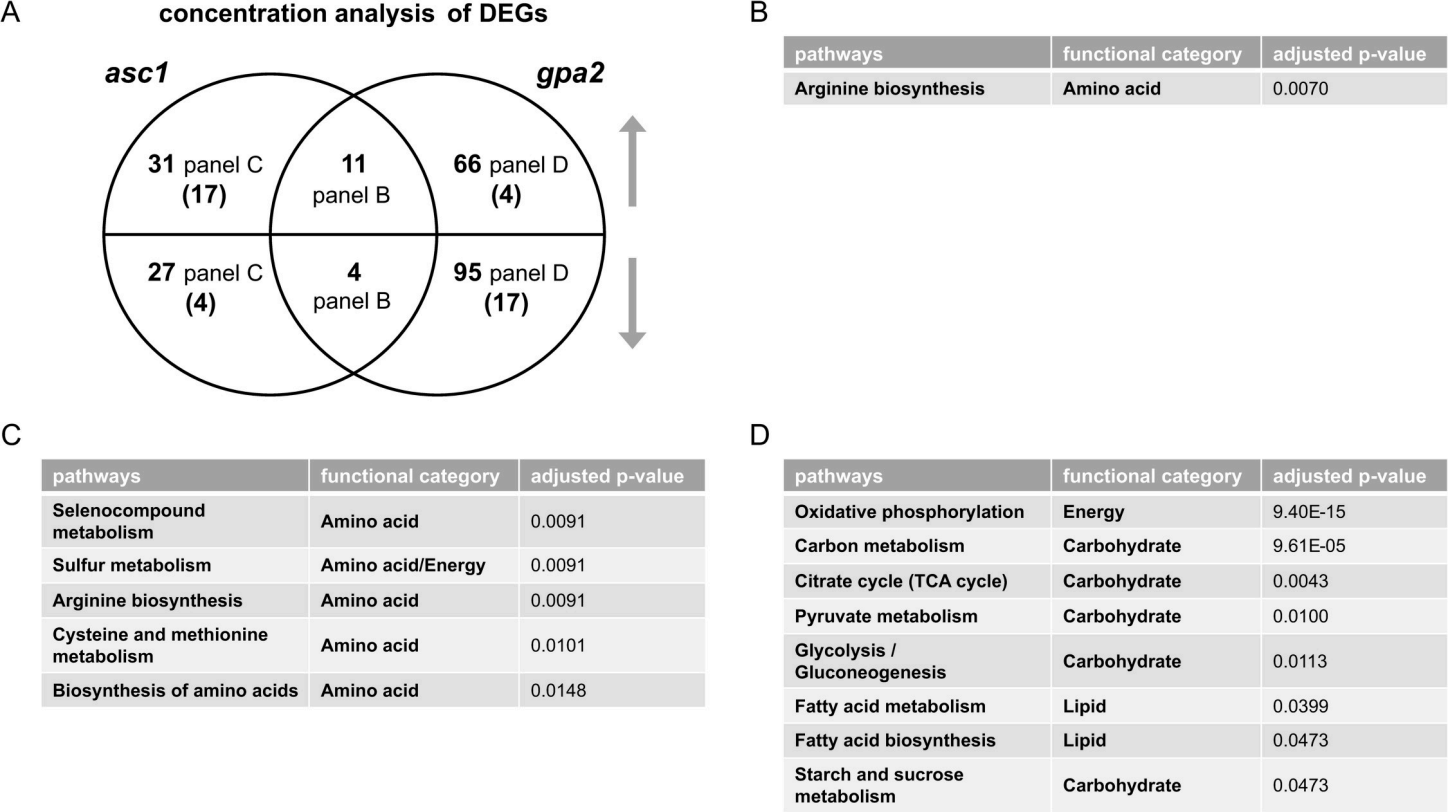
Concentration analysis of differentially expressed genes (DEGs) after glucose addition. A) Venn diagram of subsets of DEGs, for *asc1* and *gpa2* vs. wildtype, after glucose addition to 2%. Upper semicircle shows up-regulated DEGs and lower semicircle shows down-regulated DEGs. Numbers in parenthesis are shared DEGs regulated in the opposite direction, placed in the area corresponding to the direction of regulation. DEGs used for ORA analysis that are B) shared and change in the same direction; C) unique to *asc1*; D) unique to *gpa2*. Listed are all pathways and their functional categories with adjusted p-value <0.05.

According to criteria outlined above, we found 197 and 94 DEGs for *gpa2* vs. wildtype and for *asc1* vs. wildtype, respectively. Fig 2A depicts a Venn diagram, comparing the DEGs for *gpa2* vs. wildtype and for *asc1* vs. wildtype (S3 Table). We then conducted over-representation analysis (ORA) for the unique as well as shared intersects of the diagram. In contrast to GSEA, ORA requires a threshold, defined above for DEGs. In this way, we were able to compare DEGs in the two mutants and gain a detailed understanding of how they are similar and how they differ from one another. As shown in Fig 2B, the shared DEGs were over-represented for arginine biosynthesis. Arginine contributes to nitrogen balance, through urea production, and also regulates protein translation [38]. In comparison the unique DEGs were over-represented for five pathways related to amino acid metabolism (*asc1*, Fig 2C) and eight pathways linked to carbohydrate, lipid and energy metabolism (*gpa2*, Fig 2D). Most of the opposing effects were related to retrotransposon elements and are unlikely to be related to glucose metabolism or signaling. Thus, pathway analysis revealed that Asc1 mainly affects transcripts related to amino acids while Gpa2 mainly affects transcripts related to carbohydrate metabolism.

**Fig 3:**
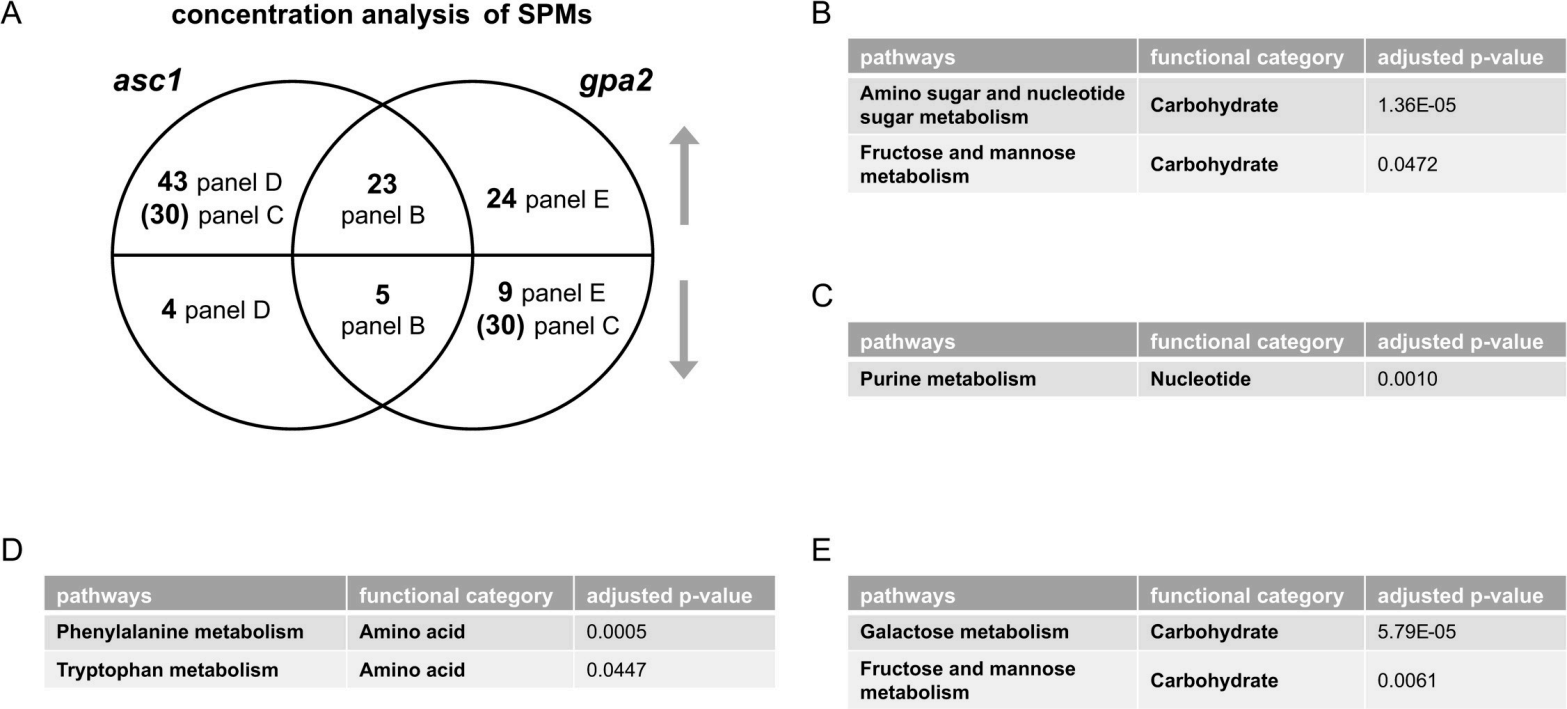
Concentration analysis of significantly perturbed metabolites (SPMs) after glucose addition. A) Venn diagram of subsets of SPMs, for *asc1* and *gpa2* vs. wildtype, after glucose addition. Upper semicircle shows up-regulated SPMs and lower semicircle shows down-regulated SPMs. Numbers in parenthesis are shared SPMs regulated in the opposite direction, placed in the area corresponding to the direction of regulation. SPMs used for ORA analysis that are B) shared and change in the same direction; C) shared and change in the opposite direction; D) unique to *asc1*; E) unique to *gpa2*. Listed are all pathways and their functional categories with adjusted p-value <0.05.

We next conducted untargeted metabolomics, followed by metabolite identification/annotations and pathway enrichment analysis, for *gpa2* and *asc1*. When compared to wildtype, *gpa2* cells were enriched in ten pathways including arginine biosynthesis as well as purine and carbohydrate metabolism (Tables 2 and S1), while *asc1* cells were enriched in four pathways including arginine biosynthesis and purine metabolism (Tables 3 and S2). Peak annotations derived from MetaboAnalystR are hereafter referred to as “metabolites”. The Venn diagram shows shared and unique significantly perturbed metabolites (SPMs, defined as those with adjusted p-value <0.05) for each mutant vs. wildtype comparison (Fig 3A and S4 Table). When comparing the two mutants, ORA revealed that those SPMs that changed in the same direction were enriched in pathways related to carbohydrate metabolism: amino sugar and nucleotide sugar metabolism, as well as fructose and mannose metabolism (Fig 3B). Because nucleotide sugars (e.g. UDP-glucose) are substrates for protein and lipid glycosylation, they may reflect preparation for new cell wall synthesis [39]. Glycosylation of the mucin Msb2 is needed to ensure signal fidelity downstream of the filamentous/invasive growth pathway [40]. SPMs perturbed in the opposite direction were enriched in purine metabolism (Fig 3C). The perturbations unique to individual mutants were over-represented for two pathways related to amino acid metabolism (*asc1*, Fig 3D) and two pathways linked to non-glucose carbohydrate metabolism (*gpa2*, Fig 3E). Contrary to what we observed for transcriptomics, pathway enrichment analysis for metabolomics revealed that more pathways were affected by the loss of *GPA2* than by the loss of *ASC1* (Tables 2 and 3). ORA analysis with subsets of SPMs corroborated the trends observed in transcriptomics. Based on these data we conclude that Asc1 mainly affects metabolites related to amino acids, Gpa2 mainly affects carbohydrate metabolism, and the two proteins have opposing effects on purine metabolism. Thus, the metabolomics data presented here mirror the transcriptomics data presented above.

To gain a deeper understanding of the functional relationship between changes in gene transcription and changes in the levels of metabolites, we employed the joint pathway analysis module in MetaboAnalystR. In this application, we input all significantly perturbed genes (DEGs) and significantly perturbed metabolites (SPMs), and queried for those over-represented in KEGG. By integrating the data in this manner, we sought to obtain more information than could be gleaned from transcriptomics and metabolomics separately. Moreover, a protein can impact a single (rate limiting) part of a metabolic pathway and have the same effect as another protein that impacts multiple components. MetaboAnalystR also provides the ‘impact score’ (S1 and S2 Tables), which weighs topological importance of components within a pathway. Because of space limitations, we only focused on the adjusted p-value for each pathway in the integration analysis.

We found that, when compared to wildtype cells, both mutants affected genes or metabolites involved in the synthesis of amino acids (cysteine, methionine, arginine, alanine, aspartic acid and glutamic acid), the metabolism of purines, galactose, amino sugars and nucleotide sugars, as well as genes/metabolites involved in ABC transporters (Tables 2 and 3 and S1 and S2). These results reveal a shared role of Asc1 and Gpa2 in regulating the metabolism of carbohydrates, as expected for any component of the glucose-sensing pathway. In addition, both subunits affected the metabolism of amino acids, particularly branches of that pathway most closely linked to the TCA cycle. Amino acids can be used for energy production in the TCA cycle, which is common when glucose is being syphoned off for anabolic processes such as making nucleotides via the pentose phosphate pathway (S3–S9 Figs). The *gpa2* strain was unique in regulating glycolysis or gluconeogenesis, oxidative phosphorylation, TCA cycle, and the pentose phosphate pathway as well as the metabolism of β-alanine, starch, sucrose, fructose and mannose (Table 2). Thus, it appears that the Gα subunit Gpa2 mainly regulates the conversion of glucose to ATP as well as general carbohydrate metabolism. In contrast, the Gβ subunit Asc1 was unique in regulating a variety of amino acids (Table 3). Thus, it appears that the Gβ subunit specifically regulates the utilization of nitrogen in part through the metabolism of arginine and other amino acids. Notably, most of the changes observed for *gpa2* and *asc1* were also observed in cells lacking their shared activator, the GPCR Gpr1 (S5 Table). More broadly, the two G protein subunits regulate distinct but complementary processes downstream of the glucose sensing receptor.

**Fig 4:**
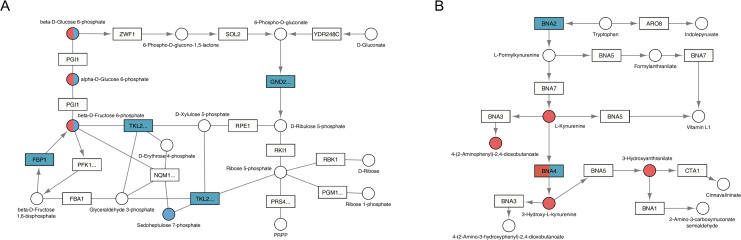
KEGG pathways regulated by *GPA2* or *ASC1* as determined by concentration analysis. The relevant part of a specific KEGG pathway is shown with genes displayed as rectangles and metabolites displayed as circles. KEGG compound name for each metabolite is labeled beside the circle. Standard gene names are labeled inside the rectangle. For enzyme complexes, the gene name for the major component is shown followed with an ellipsis. The directions of irreversible enzymatic reactions are shown by the arrows. Reversible reactions are connected by straight lines. DEGs and SPMs are highlighted in blue (*gpa2*) and red (*asc1*). Shared DEGs and SPMs are colored half blue and half red. A) as compared with *asc1*, *gpa2* affected more components in pentose phosphate pathway (functional category: carbohydrate); B) as compared with *gpa2*, *asc1* affected more components in tryptophan metabolism (functional category: amino acid).

To visualize the functional relationship of Gpa2 and Asc1, we projected the inputs of our integration analysis onto the pertinent yeast metabolic pathways in KEGG. It was evident that changes in the *gpa2* strain compared to wildtype were concentrated in regions related to glucose and ATP as well as non-glucose carbohydrates (Figs 4A and S10), while the changes in *asc1* were concentrated in various types of amino acid metabolism (Figs 4B and S11). Both mutants also impacted purine metabolism, but they did so in opposition to one another as detailed above (Fig 3C). These changes mirror the opposing effects of Gpa2 and Asc1 on cAMP. More broadly, these findings highlight the differences between the two G protein subunits and their effects downstream of the glucose-sensing receptor. Whereas Gα regulates carbohydrate utilization, Gβ regulates amino acids.

MetaboAnalystR is well suited for annotating a large number of signals. A complementary approach is to use our in-house library annotation, which includes retention time (RT), exact mass, and MS/MS library (OL) developed with data acquired for standards run under the same conditions as the study samples, as well as matching to public databases (PD), as described in Supporting Information. Metabolites are reported (S6 Table) based on the confidence in the assignment; for example OL1 is a match to the in-house library by retention time, exact mass, and MS/MS fragmentation, whereas PDa is matched to a public database by mass and experimental MS/MS. The signals identified and annotated by this method yielded pathways that mirrored those obtained using MetaboAnalystR.

In summary, our concentration analysis provides new and complementary information about glucose signaling. In particular, using an integrated transcriptomics and metabolomics approach [41,42], we were able to confirm and consolidate changes seen at the metabolic or transcriptional level alone. For example, the integration analysis confirmed results obtained at the single -omics level; that Gpa2 affects pathways related to carbohydrate utilization while Asc1 affects pathways related to amino acid metabolism. Second, integration analysis revealed information that might have been hidden using single -omics analysis methods. Only when the two datasets were combined did several important pathways meet the threshold of significance. For example, when comparing *gpa2* to wildtype, integration analysis established a substantial and statistically significant role for the Gα protein Gpa2 in glycolysis and gluconeogenesis. Of the fourteen features that emerged from our integrated analysis of this pathway, seven came from metabolomics and seven came from transcriptomics, neither of which met the threshold of significance on its own (Tables 2 and S1). Likewise, when comparing *asc1* to wildtype, integration analysis helped us to establish a specific, substantial and statistically significant role for the Gβ protein Asc1 in cysteine and methionine metabolism (Tables 3 and S2). Since the metabolic pathway is comprised of enzymes (gene products) and metabolites (enzyme products) it is noteworthy that both are regulated in a similar manner, even if the numbers obtained from each analytical method are small. Finally, our integration analysis allowed us to narrow the role for Gpa2 from very general effects on carbohydrate metabolism to a more specific role in regulating glucose and ATP. Conversely, our integration analysis allowed us to show a broader role for Asc1 in amino acid metabolism, one not limited to a specific subset of amino acids.

### Sensitivity analysis

The previous section compares the role of each signaling component in establishing transcript and metabolite concentration after glucose addition. Another way to explore the consequences of glucose sensing is to instead measure changes in response amplitude (sensitivity analysis), defined here as the difference between mutant (high minus low glucose, mutantH-mutantL) and wildtype (high minus low glucose, wtH-wtL). To put this in a biological context, a change in response amplitude reflects the role of a given component (Asc1 or Gpa2) in regulating the *relative* level of metabolites and/or genes, in response to a perturbation. A case in point is the relative change in cAMP after glucose addition, where the fold-change in its abundance is detected by the cell, regardless of the starting concentration. Differences detected using this approach are illustrated in S2B Fig.

**Fig 5:**
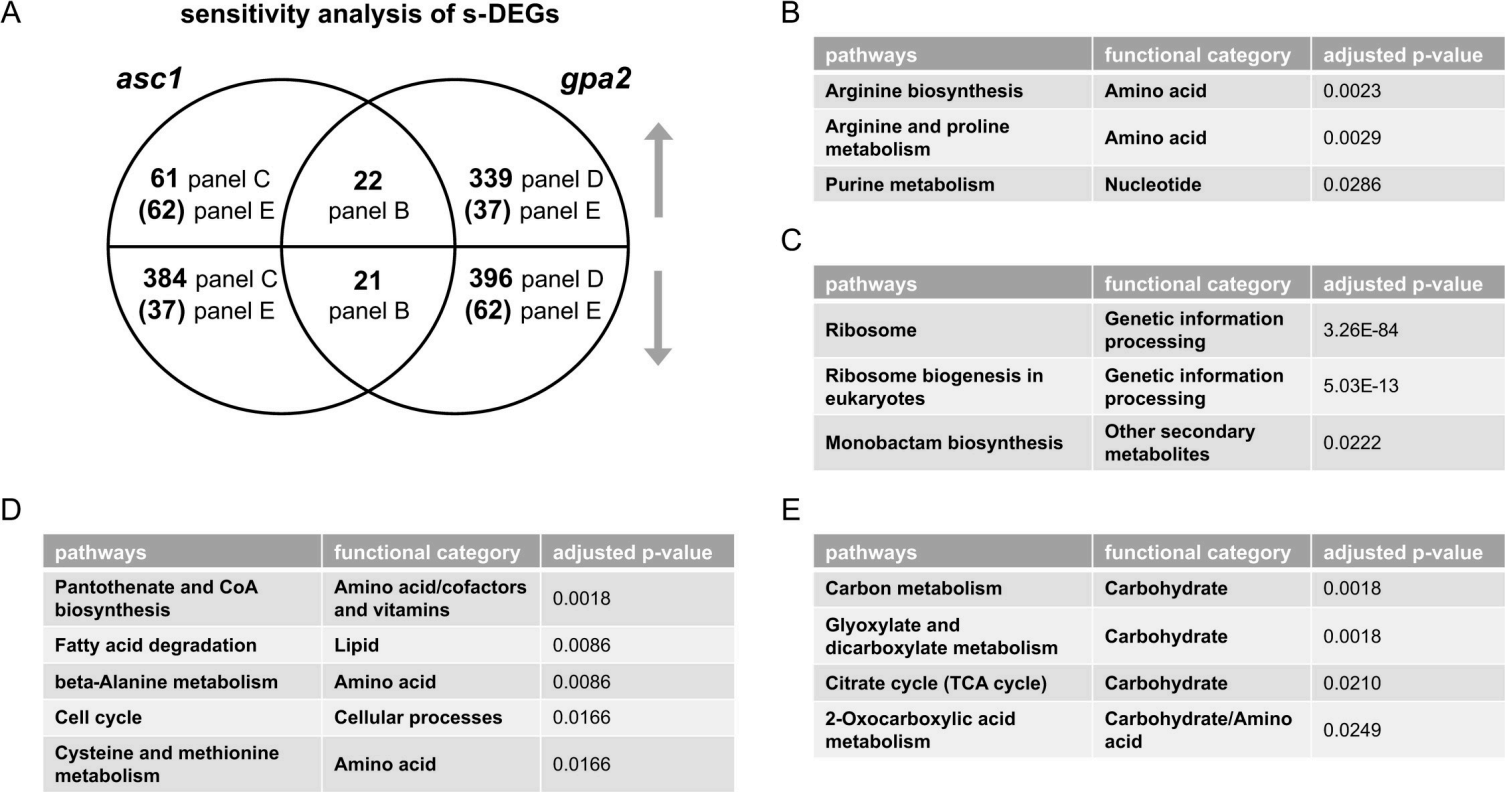
Sensitivity analysis of differentially expressed genes (s-DEGs) after glucose addition. A) Venn diagram of subsets of s-DEGs, before and after glucose addition for each mutant (mutantH-mutantL) and wildtype (wtH-wtL). Upper semicircle shows up-regulated s-DEGs and lower semicircle shows down-regulated s-DEGs. Numbers in parenthesis are shared s-DEGs regulated in the opposite direction, placed in the area corresponding to the direction of regulation. Subset of s-DEGs used for ORA analysis that are B) shared and change in the same direction; C) unique to *asc1*; D) unique to *gpa2*; E) shared and change in the opposite direction. Listed are all pathways and their functional categories with adjusted p-value <0.05.

We first compared transcriptional changes in wildtype and mutant cells, using the interaction term in DESeq2 [43]. In this analysis we found altered sensitivity for 877 and 587 genes (s-DEGs) when comparing *gpa2* with wildtype and comparing *asc1* with wildtype, respectively. In sensitivity analysis we defined s-DEGs as having altered sensitivity changes with an adjusted p-value <0.05, absolute log2 fold-change value >1 and baseMean >100. Using GSEA we determined that Gpa2 regulates the sensitivity of transcripts linked to ten pathways including N-glycan biosynthesis, carbohydrate metabolism and steroid biosynthesis (Tables 4 and S1). These changes are likely related to cell membrane and cell wall biosynthesis, leading to cell division. Asc1 regulates twelve pathways, including ribosome biogenesis and carbohydrate metabolism (Tables 5 and S2). ORA on the Venn diagram revealed that shared s-DEGs were enriched for arginine and purine metabolism (Fig 5A and 5B and S7 Table). For purine metabolism, the mutants had opposing effects for the concentration analysis (as discussed earlier) but correspondent effects for the sensitivity analysis. This suggests that purine metabolism is an important target of this glucose-sensing pathway and is congruent with the role of glucose as a precursor in purine biosynthesis. s-DEGs unique to *asc1* were over-represented for three pathways related to ribosome, ribosome biogenesis and monobactam biosynthesis (Fig 5C); s-DEGs unique to *gpa2* were over-represented for five pathways linked to amino acid and lipid metabolism (Fig 5D). These results reveal that Gpa2 uniquely affects the sensitivity of genes related to lipid metabolism (processes related to cell membrane synthesis), as well as some amino acids, while Asc1 mainly affects the sensitivity of genes related to ribosome biogenesis (processes related to new protein synthesis).

While each of the mutants had unique effects, there were an additional 99 s-DEGs regulated by both mutants but in opposite directions (S7 Table). Whereas ORA in concentration analysis revealed opposing effects for a small group of DEGs related to transposon elements, ORA in sensitivity analysis revealed opposing effects for s-DEGs related to carbohydrate metabolism (Fig 5E). For the 62 s-DEGs that were up-regulated in *asc1* and down-regulated in *gpa2*, most were due to a change in basal expression (at low glucose) (S12 Fig). For the remaining 37 s-DEGs the pattern was more complex, with mutants affecting basal expression or induced expression or both (S13 Fig).

**Fig 6:**
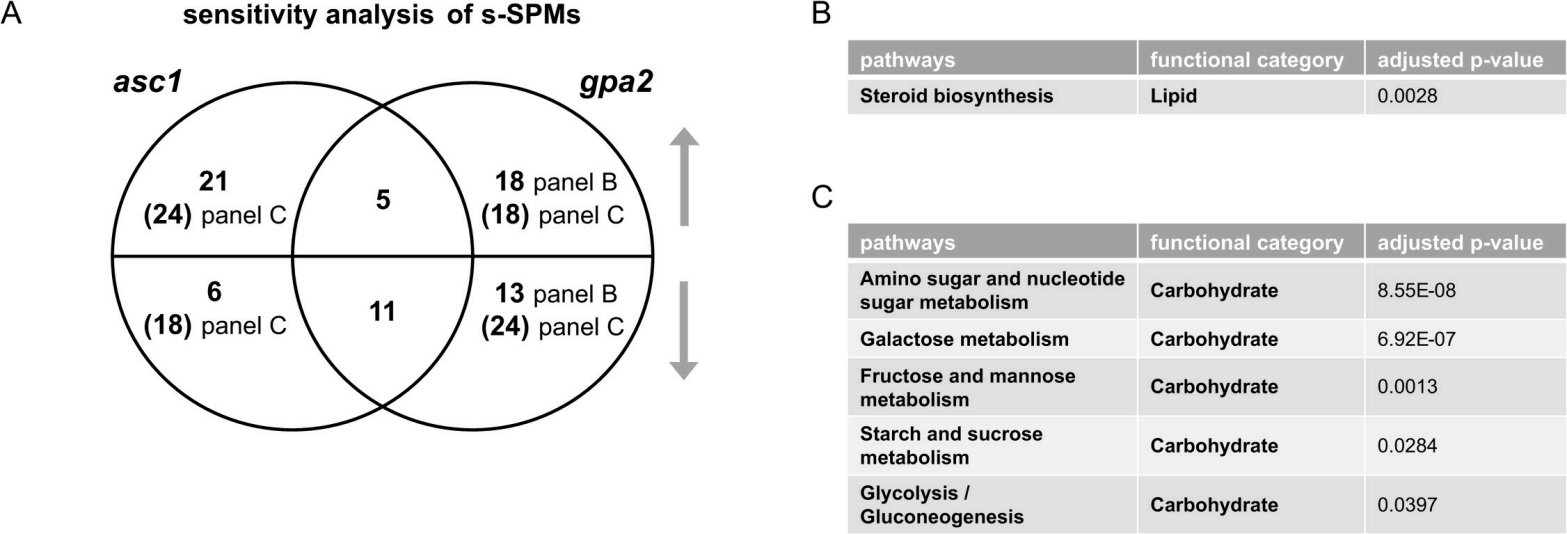
Sensitivity analysis of significantly perturbed metabolites (s-SPMs) after glucose addition. A) Venn diagram of subsets of s-SPMs, before and after glucose addition for each mutant (mutantH-mutantL) and wildtype (wtH-wtL). Upper semicircle shows up-regulated s-SPMs and lower semicircle shows down-regulated s-SPMs. Numbers in parenthesis are shared s-SPMs regulated in the opposite direction, placed in the area corresponding to the direction of regulation. Subset of s-SPMs used for ORA analysis that are B) unique to *gpa2*; C) shared and changed in the opposite direction. Shared s-SPMs changed in the same direction and s-SPMs unique to *asc1* are not over-represented in any pathway and are therefore not shown. Listed are all pathways and their functional categories with adjusted p-value <0.05.

We then performed generalized linear modeling to detect significant differences in metabolite/peak levels, comparing (wtH-wtL) and (mutantH-mutantL), followed by pathway enrichment analysis in MetaboAnalystR, as detailed above. By this approach we determined that the *gpa2* strain was primarily enriched for carbohydrate-related pathways, while *asc1* was enriched for pathways involved in lysine, cysteine, and methionine metabolism (Tables 4 and 5 and S1 and S2). Here we define s-SPMs as those with an adjusted p-value <0.05. ORA indicates that the s-SPMs unique to *gpa2* were enriched for steroid biosynthesis (Fig 6A and 6B and S8 Table). These results confirm the role of Gpa2 in lipid metabolism, as shown above. Most notably, the mutants had opposing effects on the sensitivity of an additional 42 compounds primarily related to carbohydrate metabolism (Fig 6C), consistent with the opposing effects of *gpa2* and *asc1* revealed through transcriptomics (Fig 5E).

The results above reveal important differences between the two G protein subunits, as determined by concentration and sensitivity analysis. In general, the two mutants responded in opposing ways to glucose addition, and did so at both the metabolic and transcriptional levels. Across all gene level comparisons, only arginine metabolism was affected similarly by the two mutants (Figs 2 and 5). Given the opposing roles of Asc1 and Gpa2 in other processes it is noteworthy that aspects of arginine metabolism were also affected, and in the same direction, by a mutant lacking the shared activator Gpr1 (S5 Table). More specifically, all three mutants led to significant induction of four genes (*AGP1*, *MEP1*, *DAL2*, and *GDH1*) ten minutes after glucose addition.

**Fig 7:**
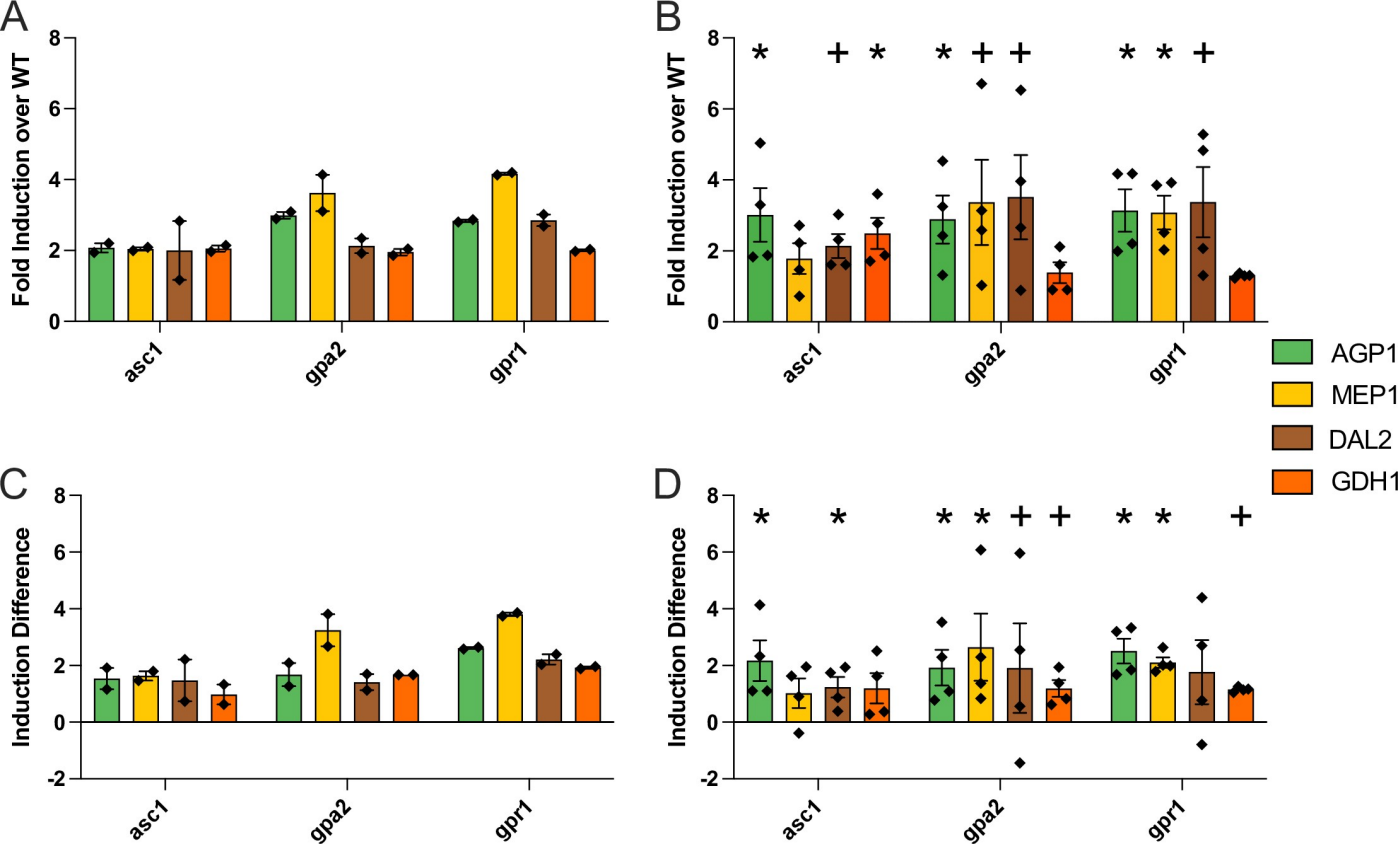
qPCR and RNA-seq analysis. A, B) Bar plots of concentration analysis results of A) RNA-seq data and B) qPCR data for *AGP1* (green), *MEP1* (yellow), *DAL2* (brown) and *GDH1* (orange). X-axes show mutants; Y-axes show fold induction over wildtype. C, D) Bar plots of sensitivity analysis results of C) RNA-seq data and D) qPCR data for *AGP1* (green), *MEP1* (yellow), *DAL2* (brown) and *GDH1* (orange). X-axes show mutants; Y-axes show change in induction between low and high glucose treatment. Error bars, standard error of the mean of biological replicates. Significance marks for qPCR analysis are as follows: p <0.05(*), p <0.15(+) by Mann-Whitney U test and adjusted with the Benjamini-Hochberg procedure.

Quantifiable changes in these gene transcripts may prove useful as reporters of glucose-mediated GPCR signaling. Accordingly, we performed qPCR to quantify the expression level of these four genes before and ten minutes after glucose addition in wildtype and all three mutants: *asc1*, *gpa2* and *gpr1*. As shown in Fig 7, all four genes responded as expected based on our RNA-seq data. Of these, the best performing transcript was *AGP1*, which was significantly induced (adjusted p<0.05) after only ten minutes, in all three mutants compared to wildtype (S9 Table), as determined by both concentration (Fig 7A and 7B) and sensitivity analysis (Fig 7C and 7D). *AGP1* encodes a broad-specificity, low-affinity amino acid permease, located at the plasma membrane [44]. Longer time courses should lead to even larger cumulative changes indicative of GPCR signaling.

We then applied integration analysis and found that both mutants affected genes or metabolites involved in the pentose phosphate pathway, as well as purine and galactose metabolism (Tables 4 and 5 and S1 and S2). The *gpa2* strain uniquely regulated a variety of cellular functions (Table 4), while *asc1* uniquely regulated ribosome function as well as arginine metabolism (Table 5). Thus, the relationship of Asc1 to amino acids and ribosome function is reflected in both concentration and sensitivity analysis. By concentration analysis, the effects of *gpa2* were primarily related to carbohydrate metabolism, while sensitivity analysis revealed a greater diversity of processes including amino acids and lipids, in addition to carbohydrates. This indicates that while Gpa2 is crucial in maintaining carbohydrate metabolism, it also interacts with genes and metabolites related to other non-carbohydrate species to ensure their proper response amplitude upon sugar addition. Taken together, it is evident that concentration and sensitivity analysis provide information that is complementary and biologically meaningful.

**Fig 8:**
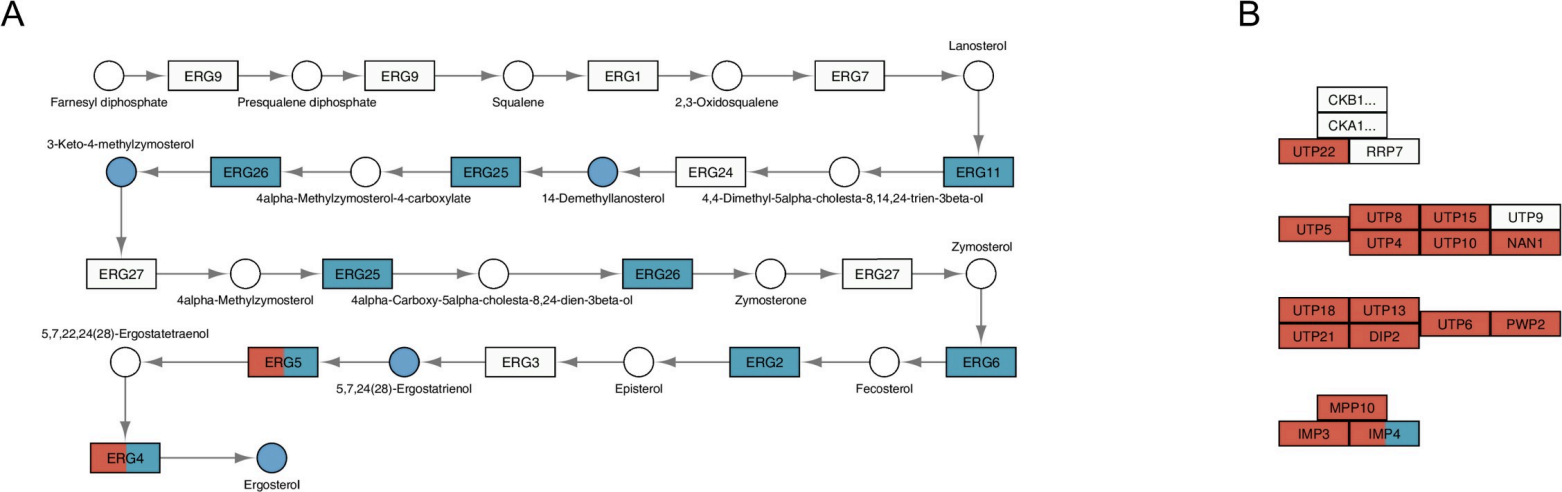
KEGG pathways regulated by *GPA2* or *ASC1* as determined by sensitivity analysis. The relevant part of a specific KEGG pathway is shown with genes displayed as rectangles and metabolites displayed as circles. KEGG compound name for each metabolite is labeled beside the circle. Standard gene names are labeled inside the rectangle. For enzyme complexes, the gene name for the major component is shown followed with an ellipsis. The directions of irreversible enzymatic reactions are shown by the arrows. Reversible reactions are connected by straight lines. s-DEGs and s-SPMs are highlighted in blue (*gpa2*) and red (*asc1*). Shared s-DEGs and s-SPMs are colored half blue and half red. A) as compared with *asc1*, *gpa2* affected more components in steroid biosynthesis (functional category: lipid); B) as compared with *gpa2*, *asc1* affected more components in ribosome biogenesis (showing 90S pre-ribosome components, functional category: genetic information processing).

**Fig 9:**
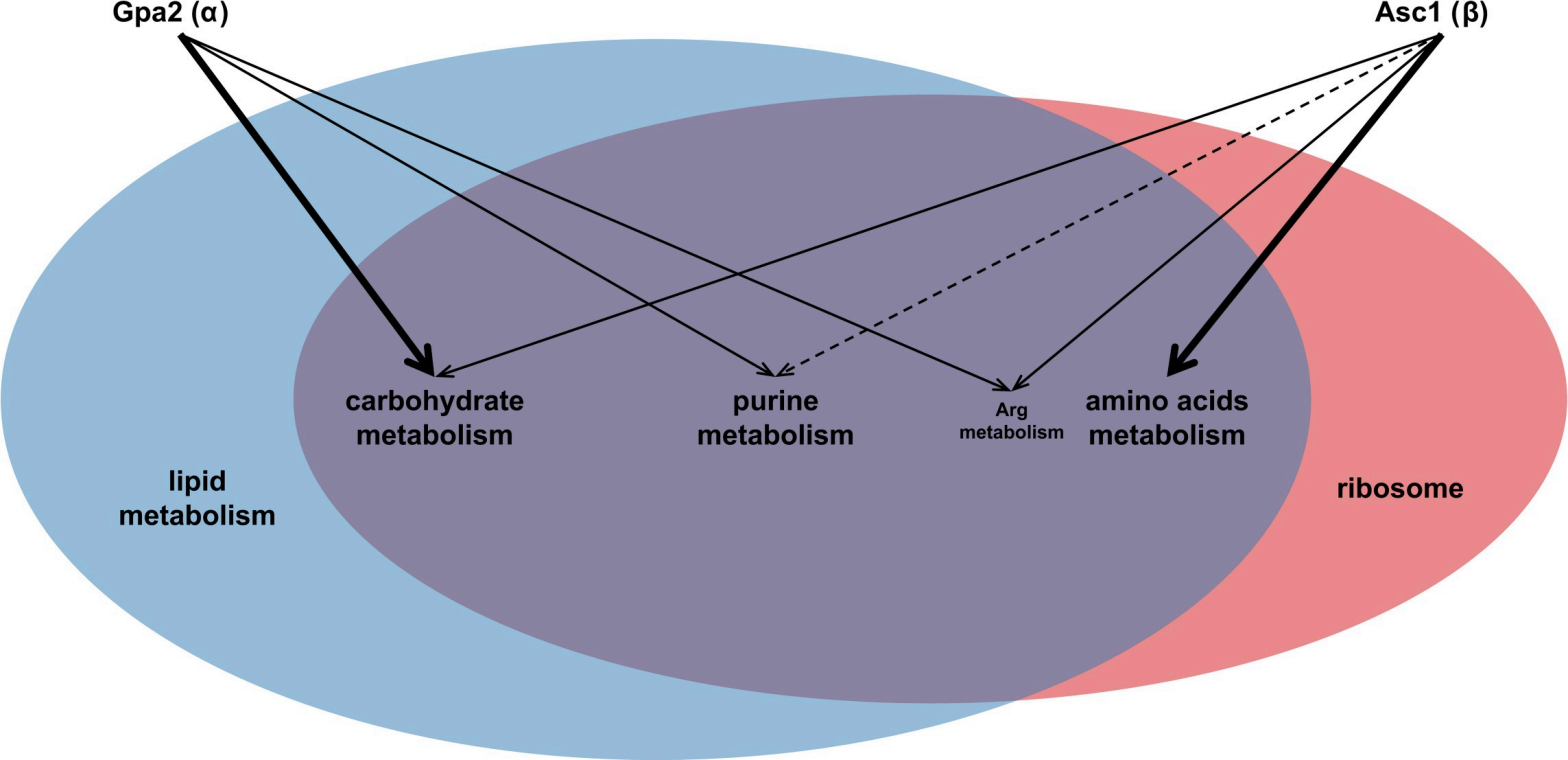
Summary of interactions. Gpa2 is primarily involved in regulating carbohydrate metabolism while Asc1 is primarily involved in amino acid metabolism, as indicated with thick lines (concentration analysis). Gpa2 and Asc1 have shared effects on arginine metabolism and opposing effects on purine metabolism, as indicated with solid and dashed lines (concentration analysis). Gpa2 regulates a greater number of gene transcripts and was particularly important in determining the amplitude of response to glucose addition, as indicated with the larger sphere (sensitivity analysis).

Finally, in order to visualize the functional relationship of Gpa2 and Asc1, we projected the inputs of our integration analysis onto pertinent yeast metabolic pathways in KEGG. It is evident that Gpa2 affects the sensitivity of a larger number of genes and metabolites, as compared with Asc1 (S14 Fig). The effects of *gpa2* were pervasive and included glucose, other carbohydrates, lipids, amino acids and purine nucleotides (Figs 8A and S14). In contrast, the effects of *asc1* centered on pentose phosphate, purine and arginine metabolism (S15 Fig). In addition, and as anticipated given its role in ribosome assembly, *asc1* uniquely affected processes related to RNA polymerase and ribosome biogenesis (Fig 8B and Table 5). These findings reveal that, although the two G protein subunits each had substantial effects by concentration analysis, the Gα subunit had by far the largest effect on sensitivity analysis. Collectively, and as summarized in Fig 9, these data indicate that Gpa2 affects the amplitude of the response to glucose, and does so for an especially large group of genes and metabolites.

## Discussion

The yeast *S*. *cerevisiae* has two functionally distinct GPCRs and two Gα proteins, but only a single canonical Gβγ. Here we considered the role of a second, “atypical”, Gβ protein Asc1 and compared its function with that of the cognate Gα protein Gpa2. Through a comprehensive analysis of glucose-dependent metabolic and transcriptional changes, we have uncovered new and common biochemical processes mediated by these two G protein subunits.

Like other Gβ proteins, Asc1 binds preferentially to Gα-GDP, slows GDP-GTP exchange [12] and has a seven-bladed propeller domain structure [24,45,46]. Like other Gβ proteins, Asc1 binds directly to the effector adenylyl cyclase; whereas Gpa2 stimulates the production of cAMP however, Asc1 has the opposite effect [12,13]. Despite their opposing effects on second messenger production, Asc1 and Gpa2 have similar effects on haploid invasive growth [12,35,36], a process by which the cells form long branching filaments and exhibit increased adherence and invasion of the substratum. This growth phenotype occurs during periods of glucose limitation [37], possibly in an attempt to direct colony expansion to sites of greater nutrient availability. The fact that the Gα and Gβ have opposing effects on cAMP but similar effects on invasive growth suggested to us that these proteins regulate processes other than adenylyl cyclase activation.

To gain a better understanding of their relative contributions to cell physiology, we undertook an integrated metabolomics and transcriptomics analysis, comparing mutants that lack one or the other of the G protein subunits. Our analysis focused on short term changes that occur in response to glucose addition, as opposed to the chronic effects resulting from deletion of *gpr1*, *gpa2* or *asc1* (while disruption of the pheromone-responsive Gα protein Gpa1 leads to permanent signaling, this behavior is not typical of other G protein systems). Correspondingly, we did not consider the effects of permanently activating Gpa2, for example by mutationally disrupting GTPase activity.

We then used several analytical methods to determine the direction and magnitude of changes in transcription and metabolism. For transcriptomics, GSEA provides an enrichment score that reflects whether a pathway is up- or down-regulated. For metabolomics, GSEA and Mummichog use different algorithms to assign directionality within a pathway, accounting for the fact that abundance of some components within that pathway might decrease while others increase. Whereas GSEA emphasizes concerted small changes Mummichog emphasizes prominent changes. Because these methods weigh the data differently, they can be difficult to reconcile with regard to pathway direction. Finally, our integration analysis (MetaboAnalyst) provides adjusted p-values, but not directionality, as an output. While our conclusions do not depend on a determination of directionality, the data provided can be used by other investigators for that purpose. Building on these findings, we are currently developing targeted assays of key metabolites, to be measured in conjunction with transcriptional reporters, in various genetic backgrounds and in response to different carbohydrate ligands, ligand concentrations, and time scales. In this way we are laying the foundation for a more comprehensive and mechanistic understanding of glucose receptor pharmacology.

As noted above, Gpa2 and Asc1 have opposing effects on cAMP production [14], as well as other metabolic processes in the cell (this work). Such competing interactions mirror those that occur in animal cells, where Gα and Gβγ proteins often have antagonistic effects on adenylyl cyclase activity [14]. While seemingly paradoxical, this design has several useful properties [47]. First, such “antagonistic bifunctionality” results in an input-output relationship that is inherently insensitive to naturally occurring fluctuations in protein abundance (“robustness”). Assuming that every Gα is partnered with a Gβ, any increase or decrease in one subunit would lead to a concomitant change in the other. A potential advantage of this arrangement is to buffer against fluctuations in the abundance of either the positive or negative regulator, which might in turn lead to substantial cell-to-cell differences in pathway activity (“noise”). Second, if the positive and negative regulators operate on different time scales they can produce outputs that are transient. This might occur if there is a delay between the inactivation of Gα (following GTP hydrolysis) and the inactivation of Gβγ (following reassociation with Gα). Third, a built-in delay can confer a property known as fold-change detection. In this scenario, the amplitude of the output is proportional to the relative difference in, rather than the absolute concentration of, the input. This can occur when the negative regulator restores the output to the pre-stimulus baseline despite sustained activation by the positive regulator. Indeed, our fold-change analysis, or sensitivity analysis, allowed us to detect phenotypes that were not evident by concentration analysis alone. All of these features are characteristic of other G protein signaling systems. An especially well-documented example is light detection by the GPCR rhodopsin, which exhibits exceptionally low noise, a transient neural output, and a wide-dynamic range of light detection [48].

Asc1 could also affect glucose signaling in other, more indirect ways. For example, others reported copurification of Asc1 with Ras1 (in *C*. *neoformans*) and a Ras GTPase-activating protein (*S*. *cerevisiae*) [49]. Together with Gpa2 [50–61], Ras1 and Ras2 activate adenylyl cyclase [52,62–64] leading to an increase in cellular cAMP [8,54,65]. Currently we are investigating novel roles of Ras1 and Ras2, using similar multi-omics approaches as those detailed above.

While many of the effects of Asc1 overlap with those of Gpa2, others appear to be unrelated to glucose sensing. Most prominently, Asc1 serves as an accessory component of the ribosome, where it is required for some protein-protein interactions within the complex and for proper protein translation [20–25,28]. It is also possible that the effects of glucose on cell metabolism are coordinated with changes in protein translation. That is to say, the protein translation machinery is made aware of changes in nutrient abundance, through the coordinated action of a shared protein Asc1. Indeed, Asc1 appears to associate with the ribosome only under glucose-rich conditions [25], and regulates a subset of transcripts related to glycolysis, respiration, oxidative stress and glucose fermentation [28].

Thus, Asc1 functions as part of two distinct multi-protein complexes, one that responds to a cell surface receptor and the other that controls protein synthesis. While seemingly incongruous, this is a phenomenon for which there is ample precedent. The recruitment of a protein that originally evolved with one function to serve a second unrelated function is an example of Darwinian exaptation, more colloquially known as “protein moonlighting” because it is analogous to a single person with two different jobs [66]. The first description of a moonlighting protein was argininosuccinate lyase, which has a well-established enzymatic function but which also serves as a structural protein responsible for the transparency of the lens and cornea [67,68]. Like Asc1, a large fraction of other proteins involved in glucose metabolism exhibit moonlighting behaviors; this includes the majority of enzymes involved in glycolysis and the tricarboxylic acid cycle [69,70]. There is also evidence for moonlighting by typical G proteins. The mammalian Gβ_2_ binds to Gα and Gγ subunits but also assembles with the ubiquitin ligase DDB1-CUL4A-ROC1, where it helps to recruit the receptor kinase GRK2 for targeted degradation [71]. Our identification of Asc1 as the Gβ for Gpa2 is like that of many other moonlighting proteins, which have been identified because of their presence in unexpected multiprotein complexes or locations.

Finally, while it has most of the functions of a typical Gβ, Asc1 has a structure that makes it unique among Gβ protein subtypes [24,45,46]. This too has precedent. Whereas Gpa1 binds to a typical Gβ (Ste4), which is necessary for pheromone-induced mating, it also associates with an atypical Gβ called Vps15, which promotes autophagy through activation of phosphatidylinositol 3-kinase [4,72]. Our x-ray structure determination revealed that Vps15 has a 7-bladed propeller domain structure analogous to that of other Gβ proteins [73], as well as a protein kinase domain of unknown function. The discovery of atypical and multifunctional Gβ proteins, including Asc1 and Vps15, suggests that the superfamily of Gβ subunits may be far larger and more complex than previously recognized [74].

It has long been appreciated that the glucose-sensing pathway in yeast employs a G protein-coupled receptor. Despite the nutritional and societal importance of glucose fermentation in yeast however, the mechanisms and molecular consequences of glucose sensing have remained obscure. By integrating transcriptomic and metabolomic measurements, we have taken a major step towards elucidating the contributions of the Gα and Gβ protein subunits in the glucose sensing pathway. We anticipate that the approaches implemented here will be useful for investigating other GPCR pathways and other moonlighting proteins, including G proteins, that participate in multiple, seemingly unrelated, biological processes.

## Methods

### Yeast strains

The prototrophic (wildtype) strain used throughout was constructed from BY4741 (MAT**a** *his3*Δ1 *leu2*Δ0 *met15*Δ0 *ura3*Δ0). *HIS3*, *LEU2*, *MET15* and *URA3* were integrated at the endogenous loci with sequence amplified by PCR from S288C strain DNA. All single mutants (*gpr1*, *gpa2*, *asc1*) were constructed by transforming the wildtype strain with corresponding sequence from the Yeast Knock-Out collection that replaces the target gene with KanMX4 [75].

### Cell preparation

All strains were cultivated in the same way and maintained at 30 ^o^C unless otherwise indicated. Cells were inoculated into Synthetic Complete (SC) (2% glucose) overnight and grown to saturation, then back diluted and kept in log phase overnight. The next morning, the culture was harvested when OD reached 1.0. For each genotype, 90 ml of culture (OD = 1.0) was split into 2 tubes (45 ml each) for later high or low glucose treatment. For each tube, cells were centrifuged and resuspended twice with SC (0.05% glucose). Cells were then resuspended into 10 ml SC (0.05% glucose) and cultivated for 1 h.

For high glucose treatment, 245 μL 65.5% glucose was added to 10 mL cell culture and for low glucose treatment, 245 μL 0.05% glucose was added to 10 mL cell culture, each for exactly 2 minutes (metabolomics) or 10 minutes (transcriptomics). The 10 minute time point was selected based on a pilot time course experiment (2, 5, 10, 15, 20, 30 and 45 minutes), showing that this is the earliest time point that captures most of the transcripts affected by the glucose treatment.

For metabolomics, each replicate consisting of 3 mL of cell culture was mixed with 45 mL cold pure methanol on dry ice. After 5 minutes, cells were centrifuged in a precooled rotor (-80°C). After discarding the supernatant, cell pellets were immediately stored at -80°C. A small aliquot of each sample was saved to manually determine cell density with a hemocytometer.

For RNA-seq, 500 μL of cell culture was aliquoted into a 1.7 mL microfuge tube and centrifuged at 1000 x *g* for 1 minute at 4°C. After discarding supernatant, the cell pellet was flash frozen by liquid nitrogen and stored at -80°C.

### Sample preparation for RNA-seq

Cell pellets stored at -80°C were resuspended with 600 μL buffer RLT 1% (v/v) 2-mercaptoethanol from the QIAGEN RNeasy Mini Kit (Cat No.: 74106) and then added into 2 mL OMNI prefilled ceramic bead tubes (SKU: 19–632). Tubes were loaded onto an OMNI Bead Mill Homogenizer (SKU:19-040E) for 3 beating cycles. For each cycle, samples were agitated at 5 m/s for 1 minute at 4 ^o^C and then cooled on ice for 3 minutes between each cycle. The resulting lysate was clarified by centrifugation at 11,000 xg and then used for total RNA extraction with QIAGEN RNeasy Mini Kit (Cat No.: 74106) with on-column DNase digestion according to manufacturer’s instructions. Extracted total RNA for each sample was checked for purity and quantified with Invitrogen Qubit 2.0 Fluorometer (Cat No.: Q32866) and Qubit RNA HS Assay kit (Cat No.: Q32855) according to manufacturer’s instructions.

### Sample preparation for metabolomics

Frozen cell pellets were resuspended with extraction reagent (8:2 methanol-water solution) to 3x10^8^ cells/mL and then transferred into 2 mL ceramic bead MagNalyser tubes. Blank samples were prepared by adding 1300 μL of extraction reagent with no cells to a MagNalyser tube with ceramic beads. Tubes were subjected to homogenization, with Bead Ruptor Elite Bead Mill Homogenizer (OMNI International) at 6.0 m/s for 40 seconds in 2 cycles at room temperature. This step was repeated twice. All samples were then centrifuged at 16,000 xg for 10 minutes at 4°C. 500 μL of the supernatant was transferred into low-bind 1.7 mL microfuge tubes. Total pools were made by combining an additional 65 μL of the supernatant from each sample and then aliquoting this mixture into low-bind 1.7 mL tubes at a volume of 500 μL. The remaining supernatant was stored at -80°C for repeat experiments if necessary. For all experimental samples, pooled samples and blanks were dried using a speedvac vacuum concentrator overnight. Dried samples were stored at -80°C.

Before LC-MS analysis, 100 μL of reconstitution buffer (95:5 water:methanol with 500 ng/mL tryptophan d-5) was added to each dried sample. All tubes were vortexed at 5000 rpm for 10 minutes and then centrifuged at room temperature at 16,000 xg for 4 minutes. Supernatant was transferred into autosampler vials for LC-MS.

### RNA library preparation

RNA libraries were prepared with Kapa stranded mRNA-seq kits, with KAPA mRNA Capture Beads (KAPA code: KK8421; Roche Cat No.: 07962207001) through the UNC High Throughput Sequencing Facility. All procedures were according to manufacturer’s instructions.

### RNA sequence analysis

Quality of raw sequence was checked with the FASTQC algorithm (http://www.bioinformatics.babraham.ac.uk/projects/fastqc/). Sequence alignment to genome indices, generated based on *Saccharomyces cerevisiae* data downloaded from Ensembl.org, was performed with the STAR algorithm [76]. Quantification on a transcriptome level was performed with the SALMON algorithm [77]. The quantified data were then analyzed with the DESeq2 package in R [78], which provides a means to determine differences in transcript abundance using a negative binomial generalized linear model [43]. Differentially Expressed Genes (DEGs for concentration analysis, s-DEGs for sensitivity analysis) are defined as having adjusted p-value <0.05, absolute log2 fold-change >1 and baseMean >100. A series of baseMean thresholds were tested, including 0, 50 and 100. The conclusion remains unchanged. Therefore, the most stringent threshold (baseMean >100, which filters out >20% of genes) was chosen for the data analysis.

PCA analysis was performed using internal PCA function of DESeq2 package with variance stabilizing transformation (vst) normalized data.

For concentration analysis, sequencing results for wildtype and all mutants after glucose addition were used as input and were analyzed with the design formula = ~batch+genotype. Here ‘batch’ is incorporated to account for batch effects of sample preparation. For sensitivity analysis, sequencing results for wildtype and all mutants before and after glucose addition were used as input and were analyzed with the design formula = ~batch+genotype+treatment+genotype:treatment. Here ‘treatment’ is equivalent to glucose addition. The interaction term ‘genotype:treatment’ is included to estimate how the response amplitude of each mutant is different from wildtype, that is (mutantH-mutantL)-(wtH-wtL).

### UHPLC high-resolution Orbitrap MS metabolomics data acquisition

Metabolomics data were acquired on a Vanquish UHPLC system coupled to a QExactive HF-X Hybrid Quadrupole-Orbitrap Mass Spectrometer (Thermo Fisher Scientific, San Jose, CA), as described previously [79]. Our UHPLC-HRMS reversed phase platform was established based on published methods [80,81]. Metabolites were separated using an HSS T3 C18 column (2.1 × 100 mm, 1.7 μm, Waters Corporation) at 50°C with binary mobile phase of water (A) and methanol (B), each containing 0.1% formic acid (v/v). The UHPLC linear gradient started from 2% B, and increased to 100% B in 16 minutes, then held for 4 minutes, with the flow rate at 400 μL/minute. The untargeted data were acquired in positive mode from 70 to 1050 m/z using the data-dependent acquisition mode.

### Metabolomics data normalization and filtration

Progenesis QI (version 2.1, Waters Corporation) was used for peak picking, alignment, and normalization as described previously [79]. Samples were randomized and run within two batches with blanks and pools interspersed at a rate of 10%. Starting from the un-normalized data for each of the batch runs, the data were filtered so as to only include signals with an average intensity fold change of 3.0 or greater in the total pools compared to the blanks. Individual samples (including pools, blanks, and study samples) were then normalized to a reference sample that was selected by Progenesis from the total pools via a function named “normalize to all”. Signals were then excluded that were significantly different between pools of batch 1 and pools of batch 2 based on an ANOVA comparison calculated in Progenesis (q <0.05). After normalization and filtration, 2397 signals passed the QC procedures and were used for further analysis.

The filtered and normalized data were mean-centered and Pareto scaled prior to conducting the unsupervised principal components analysis using the ropls R package

### In-house compound identification and annotation

Peaks were identified or annotated by Progenesis QI through matching to an in-house experimental standards library generated by acquiring data for approximately 1000 compounds under conditions identical to study samples, as well as to public databases (including HMDB, METLIN and NIST), as described previously [79]. Identifications and annotations were assigned using available data for retention time (RT), exact mass (MS), MS/MS fragmentation pattern, and isotopic ion pattern. The identification or annotation of each signal is provided in Supporting Information. Signals/metabolites that matched to the in-house experimental standards library by (a) RT, MS, and MS/MS are labeled as OL1, or (b) by RT and MS are labeled OL2a. An OL2b label was provided for signals that match by MS and MS/MS to the in-house library that were outside the retention time tolerance (± 0.5 minutes) for the standards run under identical conditions. Signals matched to public databases are labeled as PDa (MS and experimental MS/MS), PDb (MS and theoretical MS/MS), PDc (MS and isotopic similarity or adducts), and PDd (MS only) are also provided (Supporting Information).

### Transcriptomics pathway enrichment analysis and over-representation analysis

Pathway enrichment analysis for transcriptomics data was performed with ClusterProfiler package in R [30]; Log2 fold-change for each comparison (mutantH vs. wtH for concentration analysis or mutantH-mutantL vs. wtH-wtL for sensitivity analysis) was extracted from corresponding DESeq2 analysis. GSEA analysis was then performed with gseKEGG function, with organism set to ‘sce’ (*Saccharomyces cerevisiae*), permutation number set to 1000, minimal and maximal size for each analyzed geneset as 3 and 200, p-value cutoff set to 0.05, p-value adjustment method set to ‘BH’ (Benjamini-Hochberg). The KEGG sce database was used throughout, for both metabolomics and transcriptomics.

Over-representation analysis for the corresponding subsection of the Venn diagram was performed with the enrichKEGG function in ClusterProfiler package, with organism set to ‘sce’ (*Saccharomyces cerevisiae*), minimal and maximal size for each analyzed geneset as 3 and 200, p-value cutoff set to 0.05, p-value adjustment method set to ‘BH’ (Benjamini-Hochberg).

### Compound annotation, metabolic pathway enrichment analysis and over-representation analysis

Compound annotation and pathway enrichment analysis for metabolomics was performed with the MetaboAnalystR 3.0 package in R [41,42] (https://www.metaboanalyst.ca/docs/RTutorial.xhtml). For compound annotations, molecular weight tolerance (ppm) was set to 3.0, analytical mode was set to positive and retention time was included. Pathway enrichment analysis was performed with ‘integ’ module (using both Mummichog V2.0 and GSEA) with the yeast KEGG database. The p-value threshold for Mummichog was set at 0.05.

For concentration analysis, normalized peak data, from Progenesis QI for wildtype and mutants after glucose addition, were used as input for MetaboAnalystR. For sensitivity analysis, normalized peak data for wildtype and mutants before and after glucose addition were used as inputs for generalized linear model: AUC~genotype+treatment+genotype:treatment. AUC is the normalized area under curve for each peak. Treatment means before or after glucose addition. The interaction term estimated how the response amplitude of each mutant is different from wildtype, that is (mutantH-mutantL)-(wtH-wtL). The modeled p-value and t score for the interaction term associated with each peak were then used as inputs for pathway enrichment analysis. Significantly perturbed metabolites (SPMs for concentration analysis, s-SPMs for sensitivity analysis) were defined as annotations that have adjusted p-value <0.05 (FDR) from the output of MetaboAnalystR. Significantly perturbed pathways were defined as having combined p-value <0.05 (Mummichog and GSEA).

Over-representation analysis for the corresponding subsection of the Venn diagram was performed with the Enrichment Analysis module in MetaboAnalystR, with KEGG ID for each metabolites as the input. FDR adjusted p-value <0.05 was the threshold for over-represented pathways.

### Integration of transcriptomics and metabolomics data

Integration analysis was performed with the ‘joint pathway analysis’ module of MetaboAnalystR (https://www.metaboanalyst.ca/docs/RTutorial.xhtml). For both concentration and sensitivity analysis, gene input together with log2 fold-change was generated based on the corresponding DESeq2 analysis, with the threshold set as adjusted p-value <0.05, absolute log2 fold-change >1 and baseMean >100 (DEGs or s-DEGs); metabolite input together with log2 fold-change was generated based on MetaboAnalystR analysis, with the threshold set as adjusted p-value <0.05 (SPMs or s-SPMs). Integration analysis was performed on ‘all pathways’, which includes both metabolic pathways as well as gene-only pathways. Enrichment analysis was performed using ‘Hypergeometric test’. Topology measure was set to ‘Degree Centrality’. Integration method was set to ‘combine queries’, which is a tight integration method with genes and metabolites pooled into a single query and used to perform enrichment analysis within their "pooled universe". Significantly enriched pathways were defined as having FDR adjusted p-value <0.05.

### Yeast RNA extraction, DNase treatment, and reverse transcription

RNA was extracted from cells using RNeasy mini kit (Qiagen) directly as described in “Sample preparation for RNA-seq” or using hot acid phenol. Briefly, cells were resuspended in TES solution (10 mM Tris-HCl, pH 7.5; 10 mM EDTA; 0.5% SDS) and incubated with acid phenol at 65°C for one hour. Following, phenol-chloroform precipitation was used to separate the RNA, RQ1 DNase (Promega) was used to degrade any residual DNA and the samples were further purified using RNeasy mini kit. Final concentration was determined using a NanoDrop One (Thermo Scientific). To produce cDNA, 250 ng RNA was reverse transcribed with a High-Capacity cDNA Reverse Transcription Kit (Thermo Scientific) following manufacturer’s protocol.

### qPCR

qPCR primers were designed to select for short, unique regions of YCL025C (*AGP1*), YGR121C (*MEP1*), YIR029W (*DAL2*), YOR275C (*GDH1*) and reference gene YER100W (*UBC6*) and ordered from Integrated DNA Technologies, as follows:

YER100W_FWD primer: 5’ GAAGCCACGACAGGATCAAT 3’

YER100W_REV primer: 5’ ATCCCCCTCATCCAATTTTC 3’

YCL025C_FWD primer: 5’ TGGATGATGGTTTTGGGTTT 3’

YCL025C_REV primer: 5’ CTTCTGCATAACCACGAGCA 3’

YGR121C_FWD primer: 5’ GTTGGTCTGTGCTCCGGTAT 3’

YGR121C_REV primer: 5’ CTCCAAAAAGGGCATTGAAA 3’

YIR029W_FWD primer: 5’ GCTGGGAAACACGAAGACAT 3’

YIR029W_REV primer: 5’ CTTCTTCGCCCTCGTCATAG 3’

YOR375C_FWD primer: 5’ TGCCAATTGTTTCTGTTCCA 3’

YOR375C_REV primer: 5’ ACAAGTTCACGGAAGGATGG 3’

cDNA was diluted 50-fold following reverse transcription and amplified by qPCR in technical triplicate with SsoAdvanced Universal SYBR Green Supermix (Bio-Rad) according to manufacturer’s instructions modified with 45 cycles and 45 seconds anneal/extension time. Expected sequence length was verified by agarose gel electrophoresis. The threshold cycle (C_t_) was determined for each using CFX Maestro Software (BIO-RAD) and ΔΔC_t_ values were calculated in reference to wild type cells and YER100W expression levels. All values were normalized to wild type ΔΔC_t_ values. Concentration analysis was performed on cells after high glucose treatment (see “Cell preparation”) following ΔΔC_t_ analysis as described above. Sensitivity analysis was performed by comparison of ΔΔC_t_ values of high glucose treatment minus low glucose treatment. p-values were calculated using independent, non-parametric, one-tailed (with direction as determined by RNA-seq data) Mann-Whitney U tests and adjusted for multiple comparisons following the Benjamini-Hochberg Procedure.

## Supporting information

S1 Fig PCA plots. For A) transcriptomics and B) metabolomics, X-axis shows PC1 with the percentage of explained variance and Y-axis shows PC2 with the percentage of explained variance. Data are scaled as detailed in Methods. Wildtype (black), *asc1* (red), *gpa2* (blue), *gpr1* (purple). Low glucose (L, 0.05% glucose)-triangles, high glucose (H, 2% glucose)-circles.
(TIF)

S2 Fig Concentration and sensitivity analysis. Illustration of different modes of changes as captured by concentration analysis (A) and sensitivity analysis (B). The Y-axis represents either normalized gene counts or normalized peak area for metabolites. The X-axis represents different genotypes. A hypothetical wildtype is shown in black. The triangle represents measurement at low glucose and the square represents measurement at high glucose. The connecting grey line represents the response amplitude, detected by sensitivity analysis (wtH-wtL). Hypothetical mutants with increased response amplitude are colored green, while mutants with decreased response amplitude are colored red.
(TIF)

S3 Fig KEGG Metabolic Pathway Map overview. Map is color coded to delineate carbohydrate metabolism (blue), glycan biosynthesis and metabolism (cyan), amino acid metabolism (yellow), nucleotide metabolism (red), lipid metabolism (teal), metabolism of cofactors and vitamins (pink). Major species are highlighted with grey bounding box.
(PNG)

S4 Fig KEGG Metabolic Pathway Map with DEGs and SPMs of alanine, aspartate and glutamate metabolism, from *asc1* concentration analysis, highlighted in black. (PNG)

S5 Fig KEGG Metabolic Pathway Map with DEGs and SPMs of arginine biosynthesis, from *asc1* concentration analysis, highlighted in black. (PNG)

S6 Fig KEGG Metabolic Pathway Map with DEGs and SPMs of cysteine and methionine metabolism, from *asc1* concentration analysis, highlighted in black. (PNG)

S7 Fig KEGG Metabolic Pathway Map with DEGs and SPMs of alanine, aspartate and glutamate metabolism, from *gpa2* concentration analysis, highlighted in black. (PNG)

S8 Fig KEGG Metabolic Pathway Map with DEGs and SPMs of arginine biosynthesis, from *gpa2* concentration analysis, highlighted in black. (PNG)

S9 Fig KEGG Metabolic Pathway Map with DEGs and SPMs of cysteine and methionine metabolism, from *gpa2* concentration analysis, highlighted in black. (PNG)

S10 Fig Overview of DEGs and SPMs regulated by *GPA2* as determined by concentration analysis. The map is color coded as in S3 Fig. Highlighted are the DEGs (black lines) and SPMs (black dots) for *gpa2* integration analysis and gray boxes are used to delineate clusters associated with a specific pathway.
(PNG)

S11 Fig Overview of DEGs and SPMs regulated by *ASC1* as determined by concentration analysis. The map is color coded as in S3 Fig. Highlighted are the DEGs (black lines) and SPMs (black dots) for *asc1* integration analysis and gray boxes are used to delineate clusters associated with a specific pathway.
(PNG)

S12 Fig Sixty two shared DEGs affected in the opposite direction by *asc1* (up-regulated) and *gpa2* (down-regulated), by sensitivity analysis. Each gene has its own grid. Y-axis shows normalized transcript counts for low glucose (triangle), high glucose (square) in wildtype (black), *asc1* (red) and *gpa2* (blue).
(PNG)

S13 Fig Thirty-seven shared DEGs affected in the opposite direction by *asc1* (up-regulated) and *gpa2* (down-regulated), by sensitivity analysis. Each gene has its own grid. Y-axis shows normalized transcript counts for low glucose (triangle), high glucose (square) in wildtype (black), *asc1* (red) and *gpa2* (blue).
(PNG)

S14 Fig Overview of s-DEGs and s-SPMs regulated by *GPA2* as determined by sensitivity analysis. The map is color coded as in S3 Fig. Highlighted are the s-DEGs (black lines) and s-SPMs (black dots) for *gpa2* integration analysis and gray boxes are used to delineate clusters associated with a specific pathway.
(PNG)

S15 Fig Overview of s-DEGs and s-SPMs regulated by *ASC1* as determined by sensitivity analysis. The map is color coded as in S3 Fig. Highlighted are the s-DEGs (black lines) and s-SPMs (black dots) for *asc1* integration analysis and gray boxes are used to delineate clusters associated with a specific pathway.
(PNG)

S1 Table Results and statistics of transcriptomics, metabolomics and multi -omics integration for *gpa2* concentration and sensitivity analysis, each as a separate sheet. (XLSX)

S2 Table Results and statistics of transcriptomics, metabolomics and multi -omics integration for *asc1* concentration and sensitivity analysis, each as a separate sheet. (XLSX)

S3 Table List of DEGs for each subset of the Venn diagram in Fig 2A. (CSV)

S4 Table List of SPMs for each subset of the Venn diagram in Fig 3A. (CSV)

S5 Table Single- and multi-omics integration results for *gpr1* by concentration and sensitivity analysis. First block shows GSEA for transcriptomics with adjusted p-value <0.05, arranged in ascending order; second block shows MetaboAnalystR pathway enrichment analysis for metabolomics with combined p-value <0.05, arranged in ascending order; third block shows MetaboAnalystR joint pathway analysis with adjusted p-value <0.05, arranged in ascending order.
(XLSX)

S6 Table In house compound identification. (XLSX)

S7 Table List of s-DEGs for each subset of the Venn diagram in Fig 5A. (CSV)

S8 Table List of s-SPMs for each subset of the Venn diagram in Fig 6A. (CSV)

S9 Table qPCR analysis. Sheet 1: Fold induction data for concentration analysis. Sheet 2: Induction difference data for sensitivity analysis. Sheet 3: Benjamini-Hochberg corrected Mann–Whitney U test results for concentration analysis. Sheet 4: Benjamini-Hochberg corrected Mann–Whitney U test results for sensitivity analysis.
(XLSX)
