# Supplementary figures and images for: Multi-omics analysis of glucose-mediated signaling by a moonlighting Gβ protein Asc1/RACK1
Source: PLoS Genet. 2021 Jul 2;17(7):e1009640. doi: 10.1371/journal.pgen.1009640 (PMC8282090; doi:10.1371/journal.pgen.1009640)

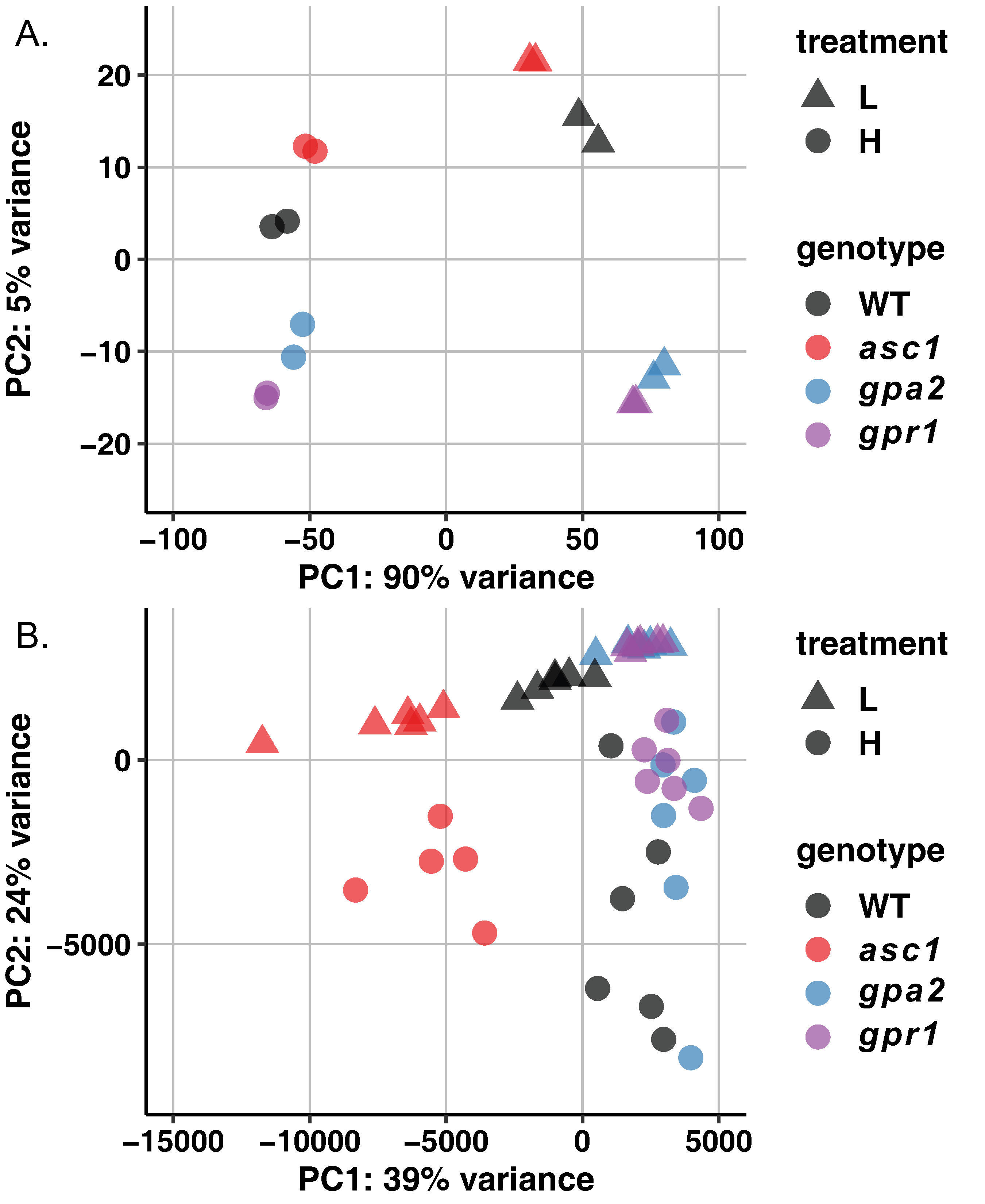

Supplement: S1 Fig — For A) transcriptomics and B) metabolomics, X-axis shows PC1 with the percentage of explained variance and Y-axis shows PC2 with the percentage of explained variance. Data are scaled as detailed in Methods. Wildtype (black), asc1 (red), gpa2 (blue), gpr1 (purple). Low glucose (L, 0.05% glucose)-triangles, high glucose (H, 2% glucose)-circles. (TIF) [file pgen.1009640.s001.tif]

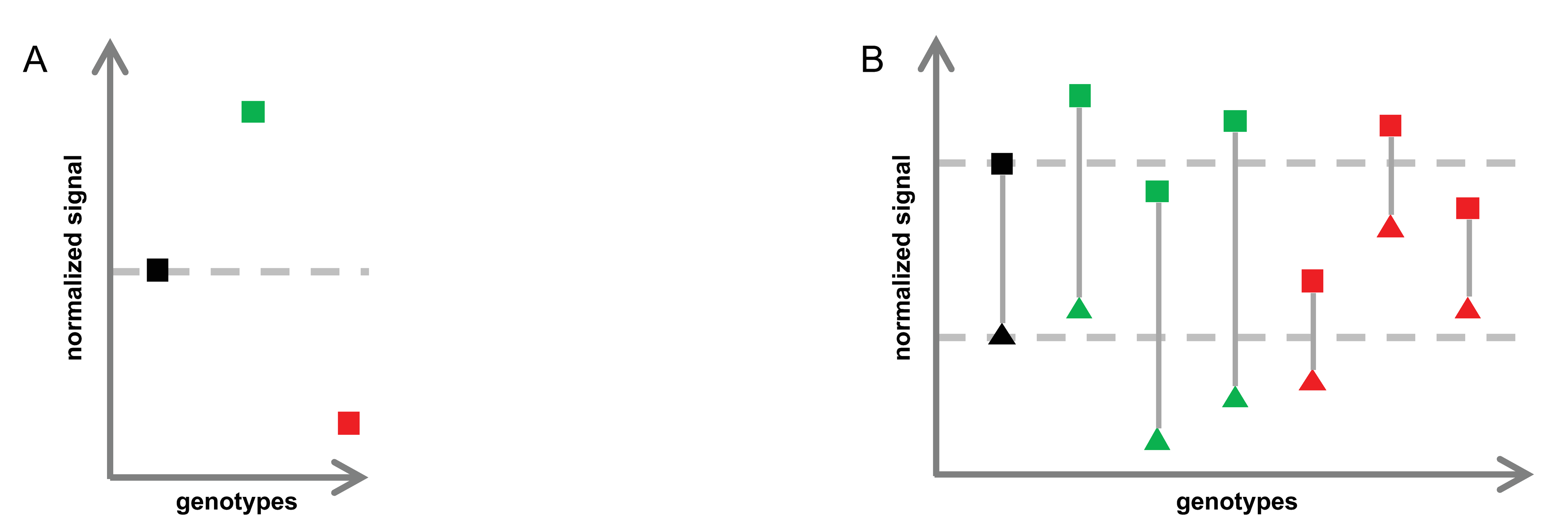

Supplement: S2 Fig — Illustration of different modes of changes as captured by concentration analysis (A) and sensitivity analysis (B). The Y-axis represents either normalized gene counts or normalized peak area for metabolites. The X-axis represents different genotypes. A hypothetical wildtype is shown in black. The triangle represents measurement at low glucose and the square represents measurement at high glucose. The connecting grey line represents the response amplitude, detected by sensitivity analysis (wtH-wtL). Hypothetical mutants with increased response amplitude are colored green, while mutants with decreased response amplitude are colored red. (TIF) [file pgen.1009640.s002.tif]

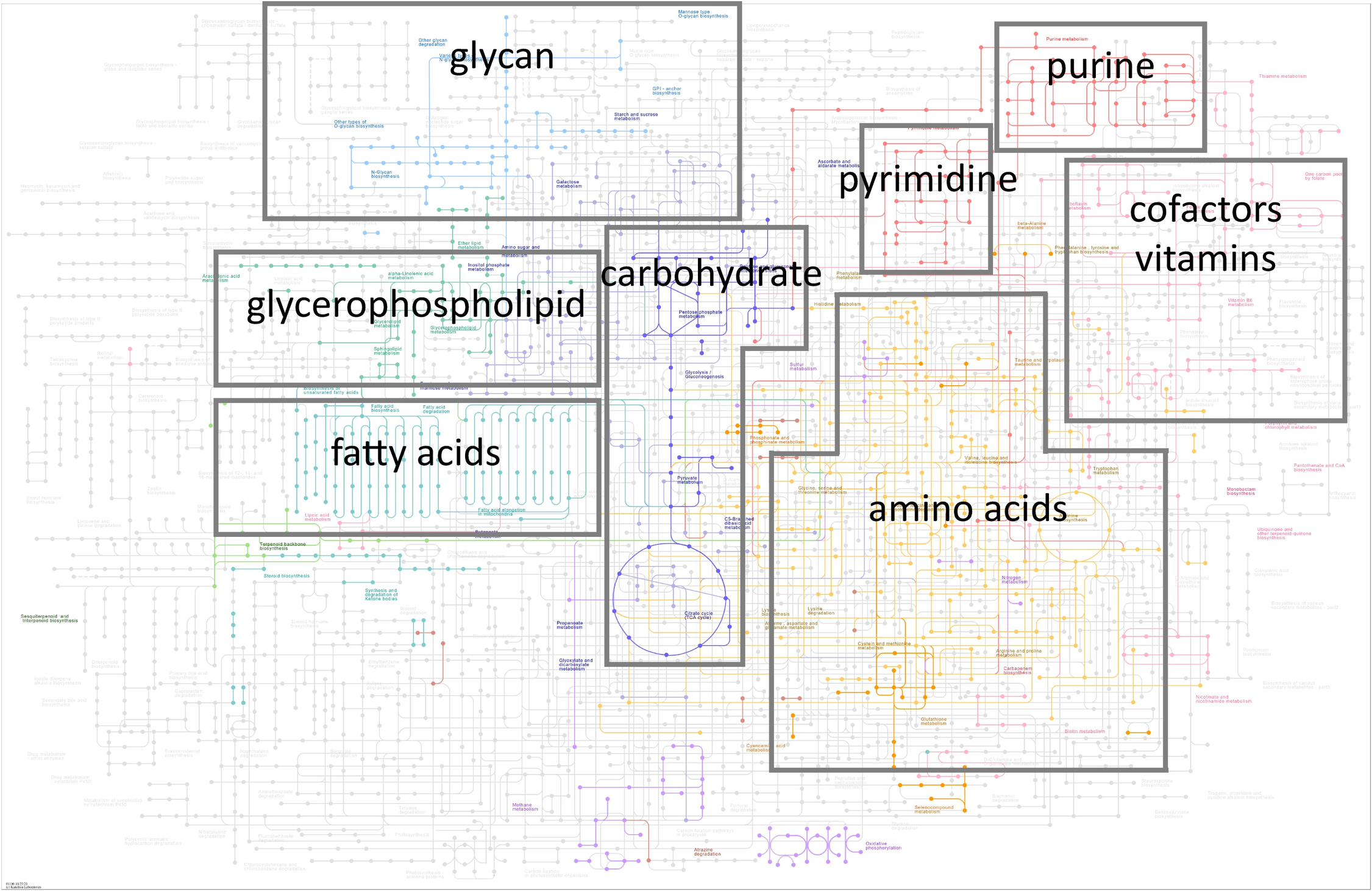

Supplement: S3 Fig — Map is color coded to delineate carbohydrate metabolism (blue), glycan biosynthesis and metabolism (cyan), amino acid metabolism (yellow), nucleotide metabolism (red), lipid metabolism (teal), metabolism of cofactors and vitamins (pink). Major species are highlighted with grey bounding box. (PNG) [file pgen.1009640.s003.png]

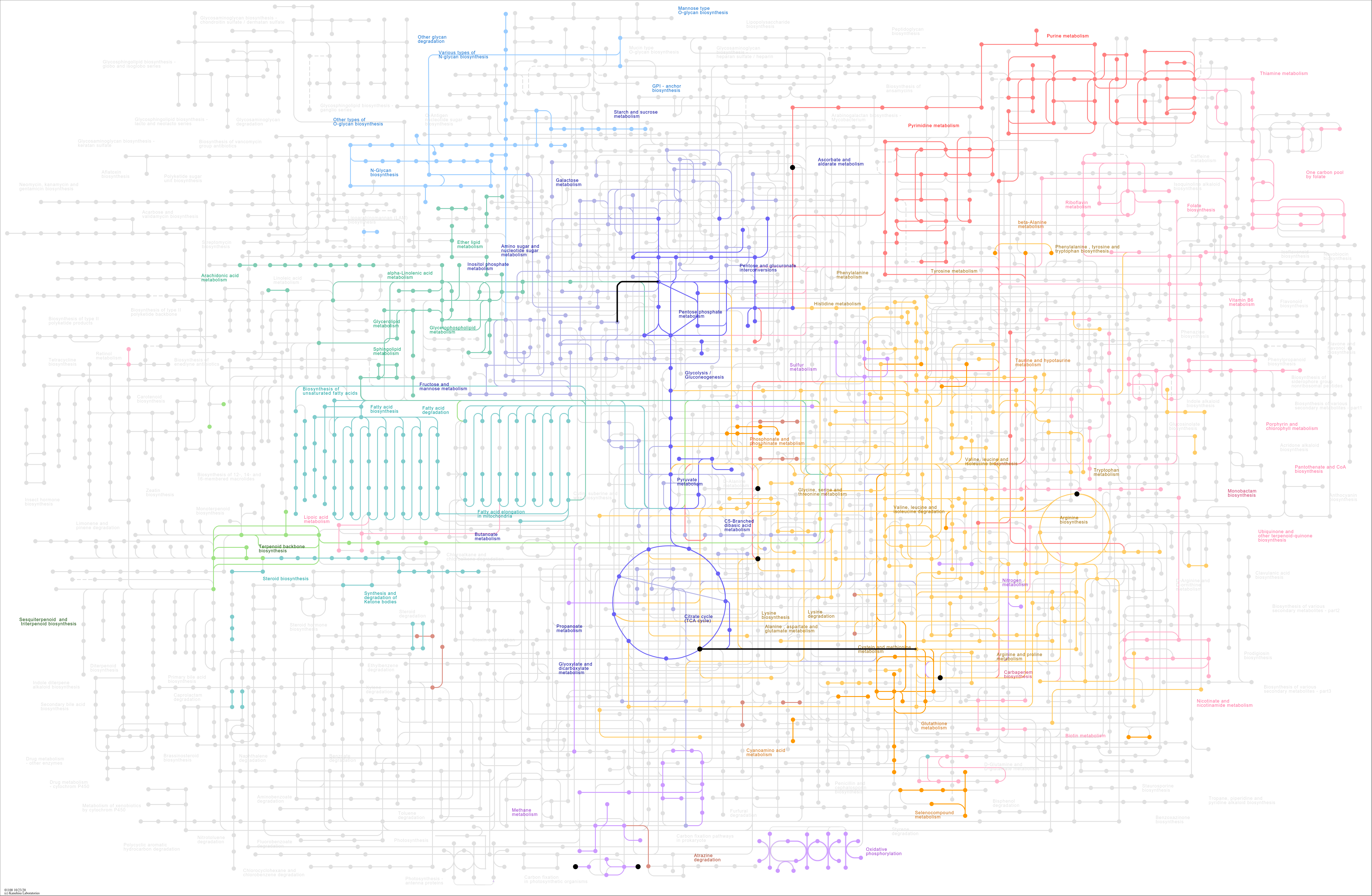

Supplement: S4 Fig — (PNG) [file pgen.1009640.s004.png]

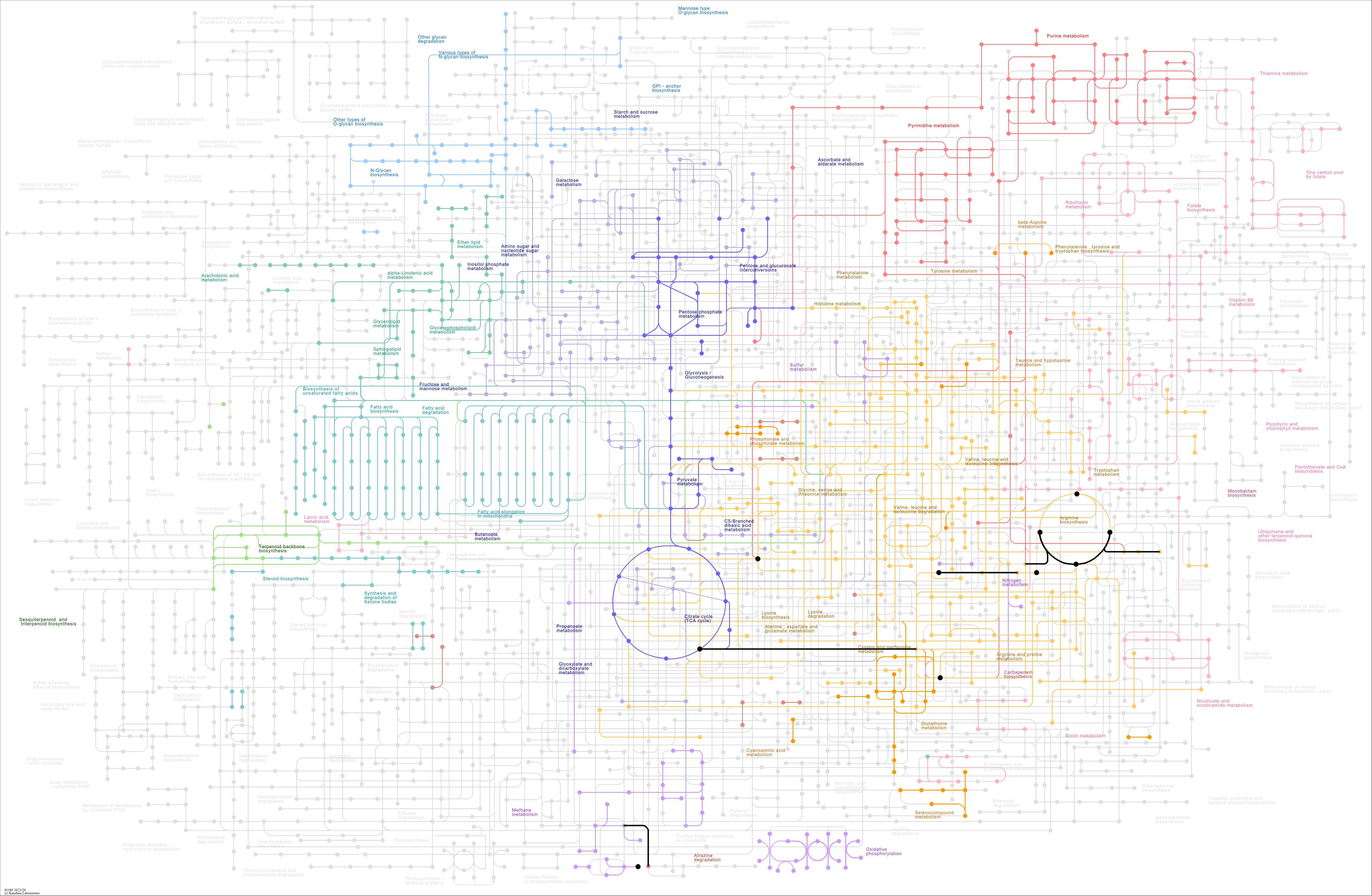

Supplement: S5 Fig — (PNG) [file pgen.1009640.s005.png]

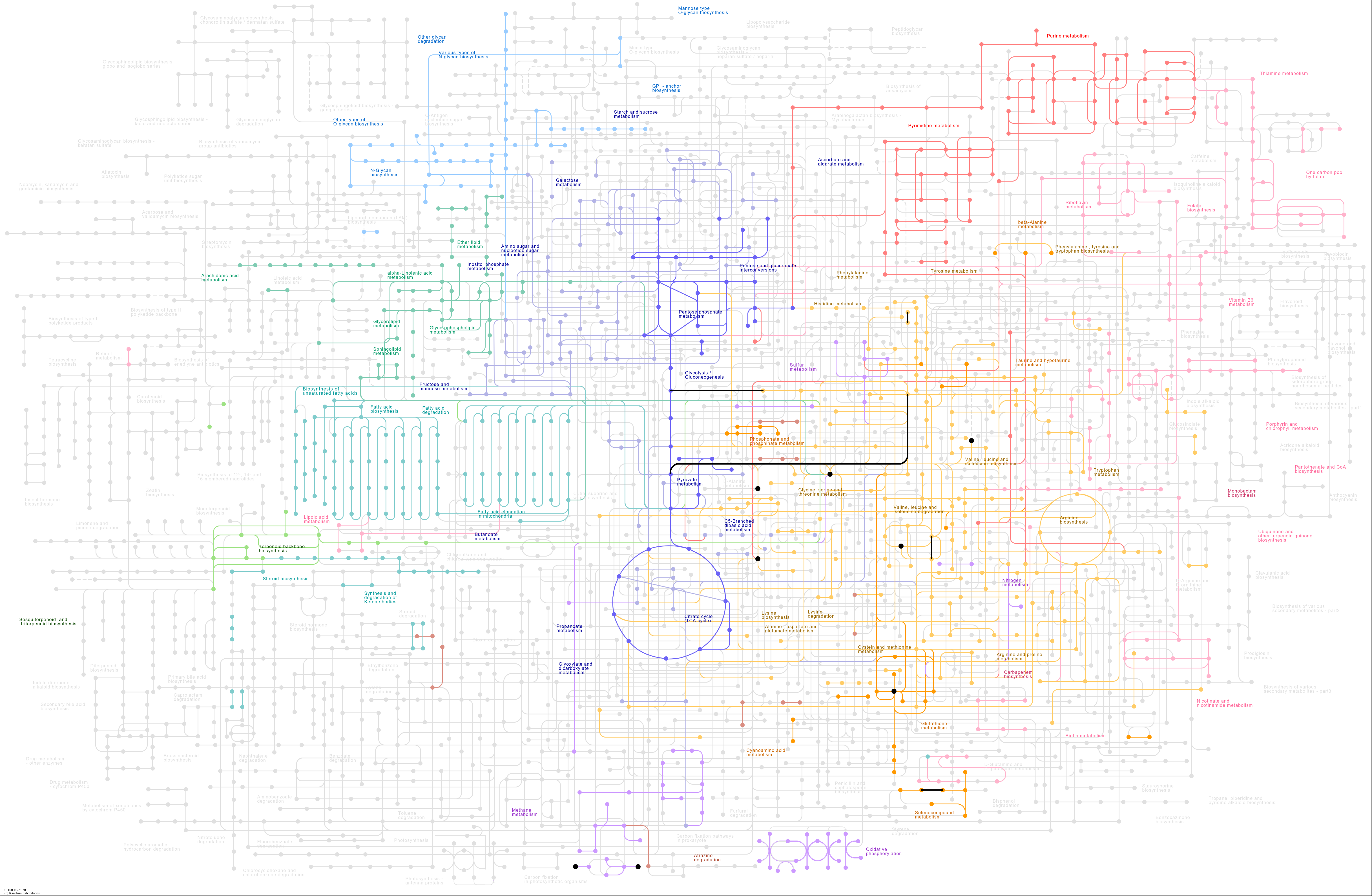

Supplement: S6 Fig — (PNG) [file pgen.1009640.s006.png]

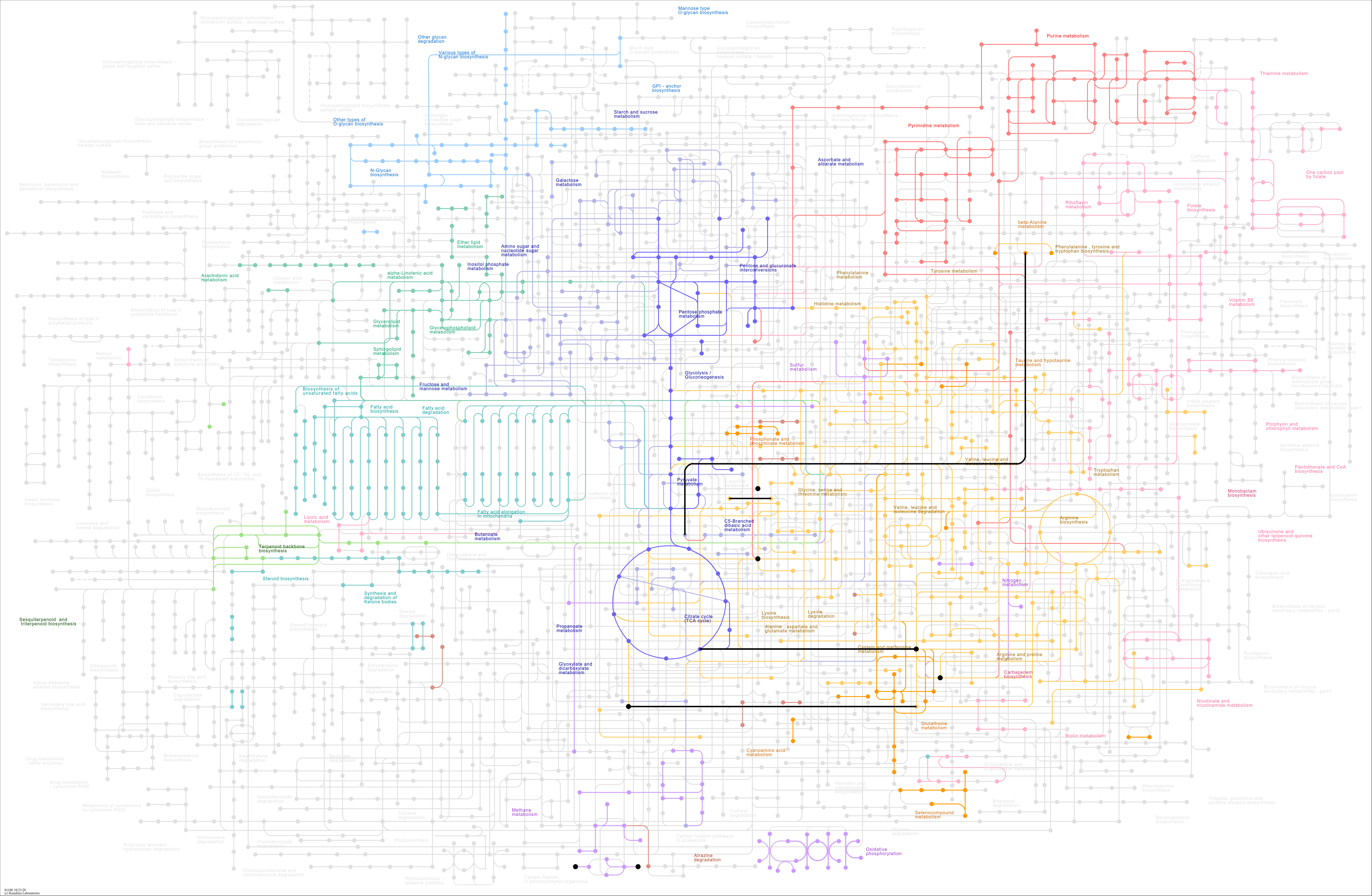

Supplement: S7 Fig — (PNG) [file pgen.1009640.s007.png]

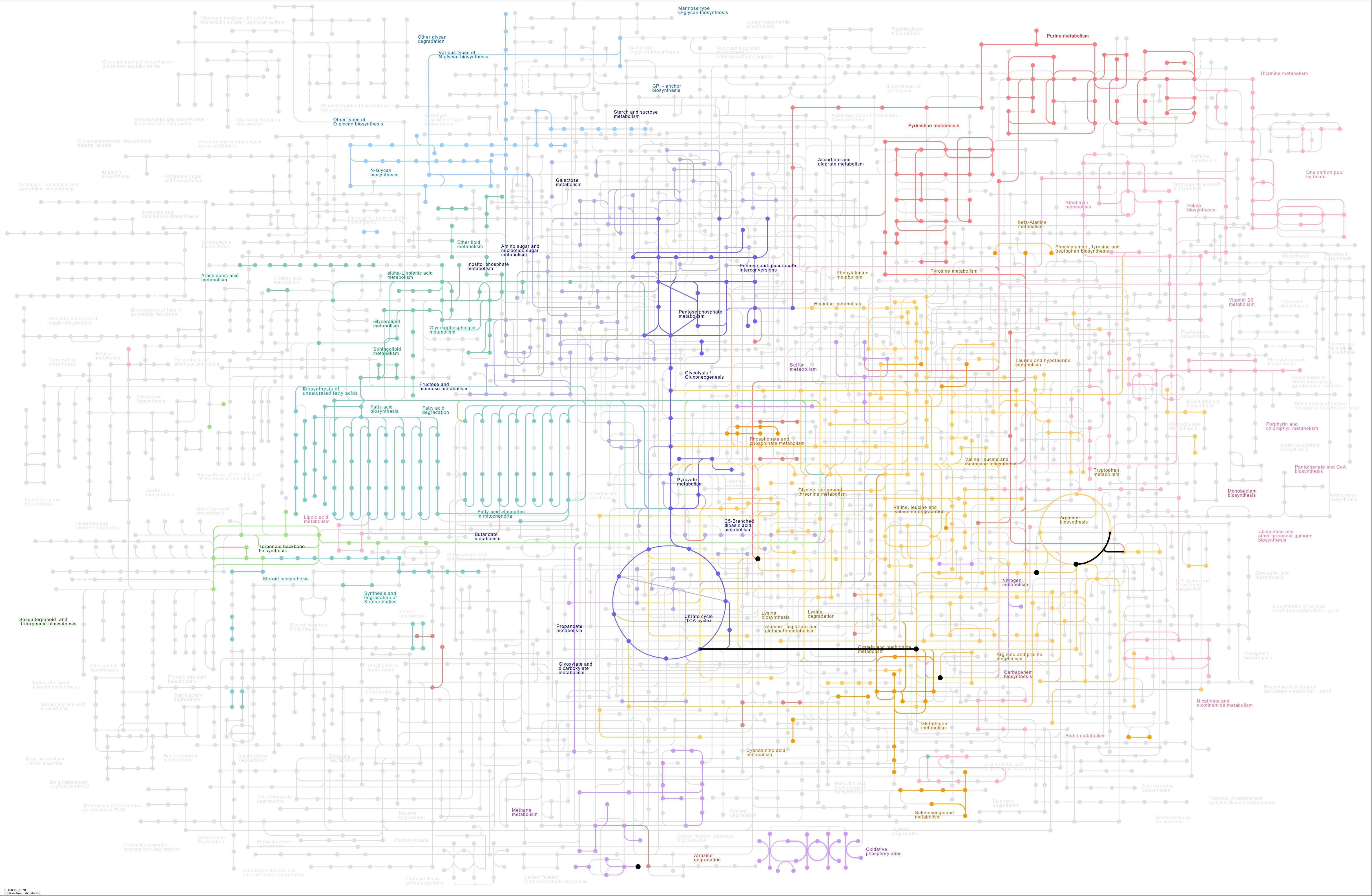

Supplement: S8 Fig — (PNG) [file pgen.1009640.s008.png]

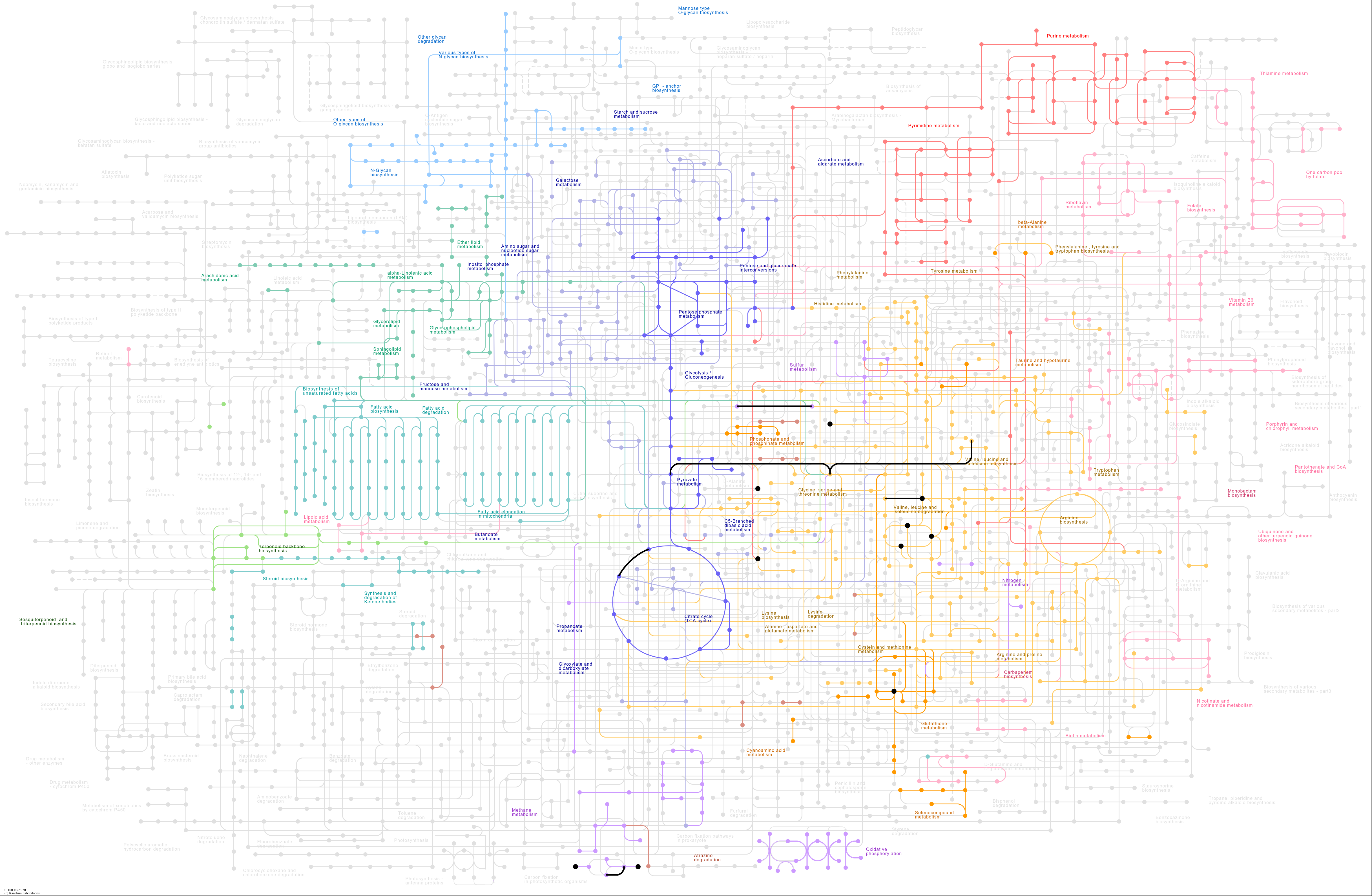

Supplement: S9 Fig — (PNG) [file pgen.1009640.s009.png]

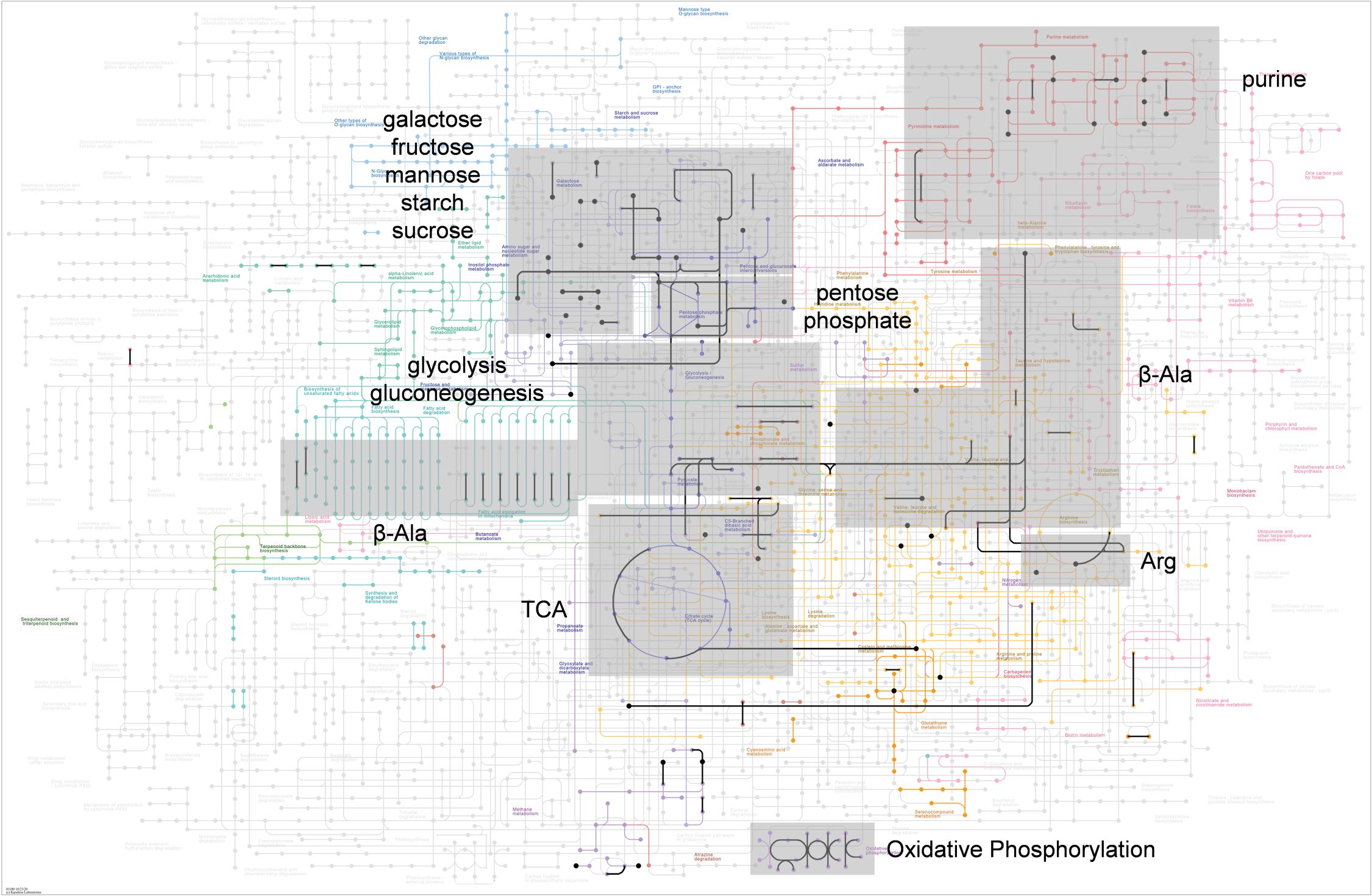

Supplement: S10 Fig — The map is color coded as in S3 Fig. Highlighted are the DEGs (black lines) and SPMs (black dots) for gpa2 integration analysis and gray boxes are used to delineate clusters associated with a specific pathway. (PNG) [file pgen.1009640.s010.png]

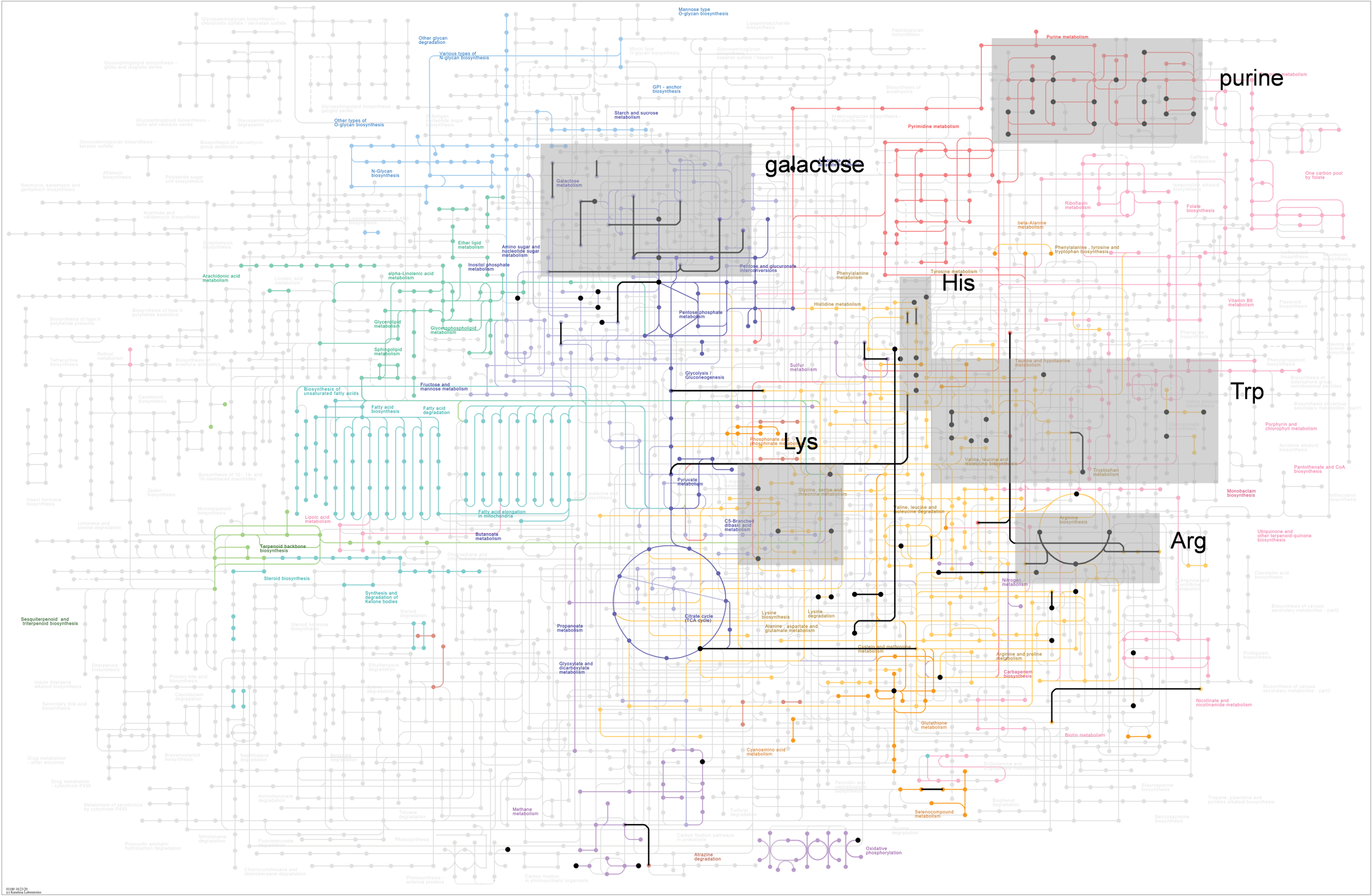

Supplement: S11 Fig — The map is color coded as in S3 Fig. Highlighted are the DEGs (black lines) and SPMs (black dots) for asc1 integration analysis and gray boxes are used to delineate clusters associated with a specific pathway. (PNG) [file pgen.1009640.s011.png]

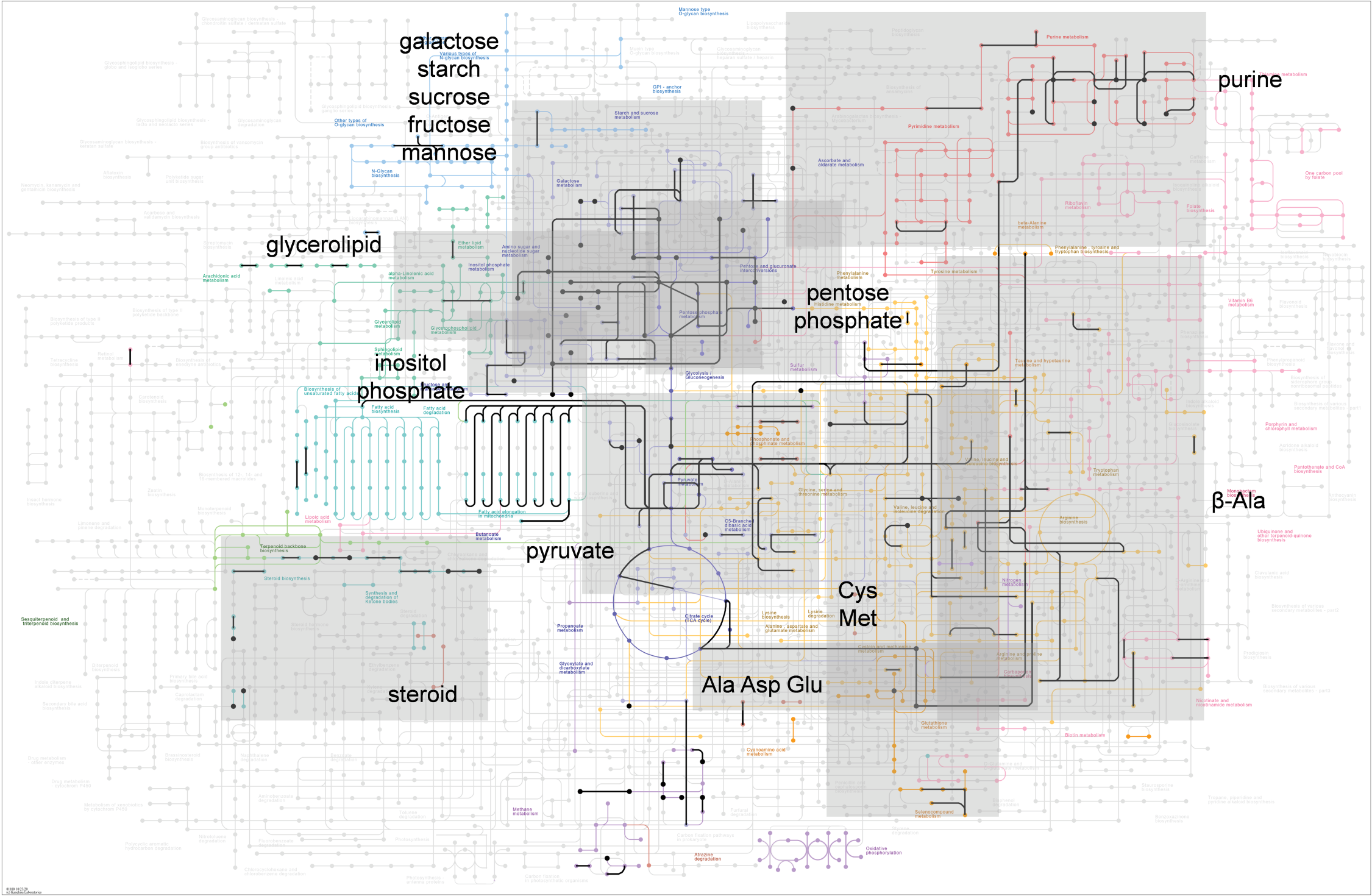

Supplement: S14 Fig — The map is color coded as in S3 Fig. Highlighted are the s-DEGs (black lines) and s-SPMs (black dots) for gpa2 integration analysis and gray boxes are used to delineate clusters associated with a specific pathway. (PNG) [file pgen.1009640.s014.png]

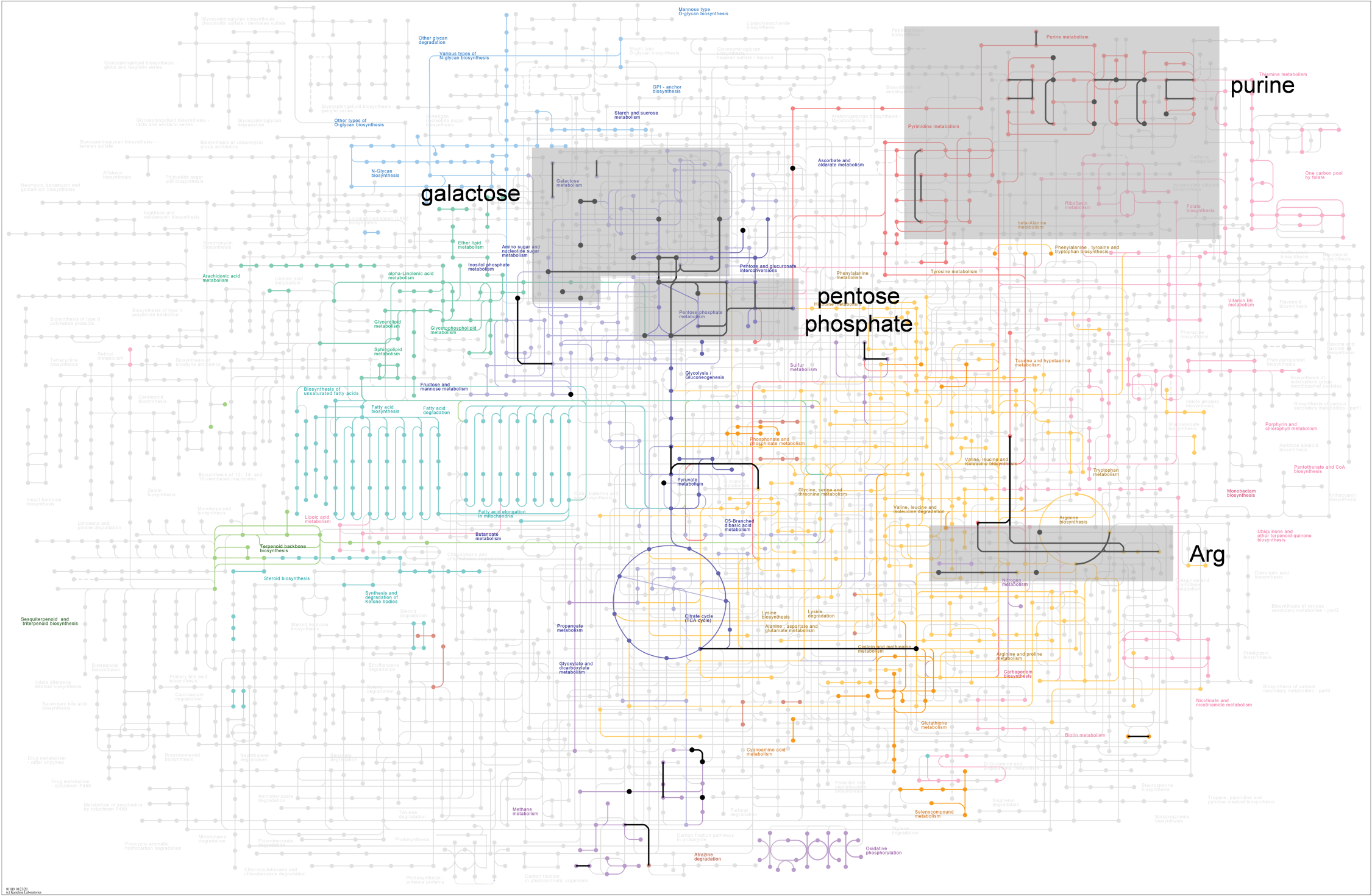

Supplement: S15 Fig — The map is color coded as in S3 Fig. Highlighted are the s-DEGs (black lines) and s-SPMs (black dots) for asc1 integration analysis and gray boxes are used to delineate clusters associated with a specific pathway. (PNG) [file pgen.1009640.s015.png]
